# Changing the home visiting research paradigm: models’ perspectives on behavioral pathways and intervention techniques to promote good birth outcomes

**DOI:** 10.1186/s12889-022-13010-5

**Published:** 2022-05-21

**Authors:** Anne K. Duggan, Kelly M. Bower, Ciara Z. Spinosa, Kay O’Neill, Deborah Daro, Kathryn Harding, Allison Ingalls, Allison Kemner, Crista Marchesseault, William Thorland

**Affiliations:** 1 Johns Hopkins Bloomberg School of Public Health, 615 N. Wolfe Street, Baltimore, MD 21205 USA; 2 Johns Hopkins School of Nursing, 525 N. Wolfe Street, Baltimore, MD 21205 USA; 3 Chapin Hall at the University of Chicago, 1313 E. 60th Street, Chicago, IL 60637 USA; 4 Healthy Families America at Prevent Child Abuse America, 228 S. Wabash, 10th Floor, Chicago, IL 60604 USA; 5 Department of International Health, Center for American Indian Health, Johns Hopkins Bloomberg School of Public Health, 415 N. Washington Street, Baltimore, MD 21231 USA; 6 Parents As Teachers National Center, 2228 Ball Drive, St. Louis, MO 63146 USA; 7 Minding the Baby National Office: Yale Child Study Center & Yale School of Nursing, Yale University, P.O. Box 208056, New Haven, CT 06520 USA; 8 Nurse-Family Partnership, National Service Office, 1900 Grant Street, Suite 400, Denver, CO 80203 USA

**Keywords:** Precision services, Home visiting, Intervention techniques, Birth outcomes

## Abstract

**Background:**

The US is scaling up evidence-based home visiting to promote health equity in expectant families and families with young children. Persistently small average effects for full models argue for a new research paradigm to understand what interventions within models work best, for which families, in which contexts, why, and how. Historically, the complexity and proprietary nature of most evidence-based models have been barriers to such research. To address this, stakeholders are building the Precision Paradigm, a common framework and language to define and test interventions and their mediators and moderators. This observational study used portions of an early version of the Precision Paradigm to describe models’ intended behavioral pathways to good birth outcomes and their stance on home visitors’ use of specific intervention technique categories to promote families’ progress along intended pathways.

**Methods:**

Five evidence-based home visiting models participated. Model representatives independently completed three structured surveys focused on 41 potential behavioral pathways to good birth outcomes, and 23 behavior change technique categories. Survey data were used to describe and compare models’ intended behavioral pathways, explicit endorsement of behavior change technique categories, expectations for home visitors’ relative emphasis in using endorsed technique categories, and consistency in endorsing technique categories across intended pathways.

**Results:**

Models differed substantially in nearly all respects: their intended pathways to good birth outcomes (range 16–41); the number of technique categories they endorsed in any intended pathway (range 12–23); the mean number of technique categories they endorsed per intended pathway (range 1.5–20.0); and their consistency in endorsing technique categories across intended pathways (22%-100% consistency). Models were similar in rating nearly all behavior change technique categories as at least compatible with their model, even if not explicitly endorsed.

**Conclusions:**

Models successfully used components of the Precision Paradigm to define and differentiate their intended behavioral pathways and their expectations for home visitors’ use of specific technique categories to promote family progress on intended pathways. Use of the Precision Paradigm can accelerate innovative cross-model research to describe current models and to learn which interventions within home visiting work best for which families, in which contexts, why and how.

**Supplementary Information:**

The online version contains supplementary material available at 10.1186/s12889-022-13010-5.

## Contributions to the literature

- The prevailing research paradigm for home visiting has been randomized trials of full models to estimate average effects on outcomes.
- Persistent challenges in engaging and positively impacting families calls for a new paradigm to answer, *What works best, for which families, in which contexts, why and how?*
- In this proof-of-concept project, home visiting models successfully used our structured approach to identify their intended behavioral pathways and stance on home visitors’ use of specific behavior change techniques to promote good birth outcomes.
- A broad range of intervention techniques is compatible with existing evidence-based models.
- We continue to work with stakeholders to refine and apply the Precision Paradigm with other outcomes and stakeholder groups to assess existing models’ coherence and clarity and existing implementation system adequacy, and to design and test new interventions compatible with existing evidence-based models.

## Background

Prenatal and early childhood home visiting is a public health prevention strategy for expectant families and families with children birth to five years. In the US and internationally, it is a key strategy to reach and promote health equity among underserved families facing multiple adversities such as poverty, poor access to healthcare, systemic racism, histories of trauma, parental behavioral health issues, and lack of parenting expertise.

In 2010 and again in 2018, Congress authorized federal investment to scale up evidence-based home visiting through the Maternal, Infant and Early Childhood Home Visiting Program (MIECHV Program) [1, 2]. The MIECHV Program is administered by the Health Resources and Services Administration and the Administration for Children and Families. Most funding is awarded to states and territories to expand evidence-based home visiting availability, usually through contracts with community-based organizations and local implementing agencies. Some award funding is dedicated to tribes, tribal organizations, and urban Indian organizations to develop, implement, and evaluate home visiting programs in American Indian and Alaska Native communities.

Many of the 19 home visiting models designated as evidence-based for MIECHV Program purposes are comprehensive, aiming to promote a broad range of outcomes through frequent visits over many months or a few years [3]. All models aim to promote child well-being. They vary in the parenting, family functioning, and other factors they address to promote child well-being, and in their theories of change. All models provide direct service; most also link families with community resources.

Population-level change through scale up of evidence-based interventions requires reaching intended families, engaging them, and implementing services with fidelity [4]. The MIECHV Program’s national evaluation—an 88-site, cross-model study of family reach and engagement, implementation and impact—provides valuable insights on these aspects of the scale up of evidence-based models of home visiting. The implementation study found that local programs reached intended families but failed to engage many of them [5]. It also found that local programs’ implementation systems were uneven, providing stronger support for staff to address parenting directly than to address its contributing factors. Study results showed that this unevenness was reflected in service delivery [5]. Home visiting significantly improved several postnatal confirmatory outcomes but with very small average effects [6, 7].

The concordance of these results with those of earlier research reveals weaknesses of the traditional home visiting research paradigm for refining models and implementation systems to advance the field. For the past several decades, the field’s traditional research paradigm has been to use randomized trials to estimate average effects of full home visiting models. Research reports have typically described models’ theories of change and model components in broad terms, assessed fidelity and family engagement only lightly, if at all, and restricted testing for subgroup impacts to post hoc analyses. This research paradigm has been essential for building the evidence base for full models but has been inadequate to inform strategies to strengthen family engagement, fidelity of implementation, and impacts on outcomes across models. It has been inadequate as well for advancing the field’s understanding of home visiting’s core components [8].

Open science can improve research reporting to promote replication of results and effective scale up [9], but only insofar as home visiting embraces a research paradigm that can answer the question, *What works best, for which families, in which contexts, why and how?* [10, 11] For home visiting, this requires: 1) specifying and testing interventions within home visiting rather than focusing only on full models; 2) going beyond estimating average effects on outcomes by testing mediators and moderators; and 3) using innovative designs to achieve actionable results and optimize impacts across varied contexts. Such an approach, if coupled with a standard framework and language across home visiting models and studies, can accelerate improvements in models and implementation systems, and the confidence of decision-makers who rely on research to guide their policy and programming decision-making.

Home visiting models are complex interventions, but advancement of the field now requires a more granular approach in defining and testing home visiting’s component parts [12]. Recognition of the benefits of a granular approach has motivated a shift in this direction beyond home visiting. Examples include the distillation of approaches used within manualized mental health treatments for adolescents [13], the deconstruction of interventions to address obesity into their component behavior change techniques [14], identification of the content and relational components of motivational interviewing [15], and analysis of provider–client interactions into task-, social-emotional-, and activation-focused strategies [16]. Pioneering researchers use different strategies to organize the range of potential behavior change components of interventions. One option is hierarchical taxonomies of highly standardized terms to facilitate systematic reviews and meta-analytic studies [17]. Another is to organize theory-based intervention techniques by the mechanisms of action they are likely to influence, to facilitate intervention development [18].

Several features of home visiting complicate shifting the research paradigm. Most models are proprietary; this can inhibit sharing of detailed information on interventions. Many models are complex and comprehensive, comprised of interwoven interventions whose intended independent and interactive effects might not yet be fully and explicitly conceptualized. Many comprehensive models were designed with the long view in mind; focusing on short-term target behaviors requires changing focus. Some models are grounded more in explanatory theories than in theories of behavior change.

The Home Visiting Applied Research Collaborative (HARC) is a national research and development platform charged with shifting the home visiting paradigm [10, 11, 19, 20]. HARC brings together stakeholders – model developers, researchers, administrators, front line staff and families – to build and use a common research framework and language to advance precision.

**Fig. 1:**
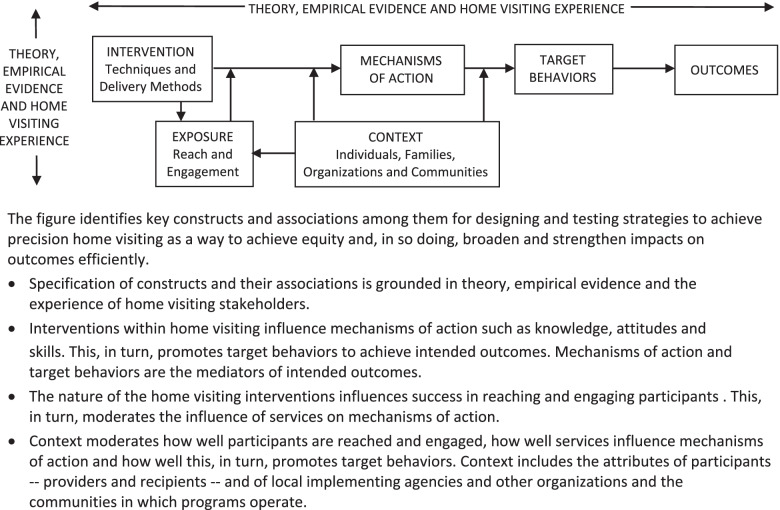
Home visiting Precision Paradigm (adapted from the Human Behaviour Change Project [21])

Figure 1 illustrates key components of the evolving framework, called the Precision Paradigm. It is based on the ontology of the Human Behaviour Change Project, arguably the most widely disseminated ontology for behavioral health interventions [21]. We chose this as the basis for the Precision Paradigm because it is applicable for intervention research across diverse outcomes and contexts, just as home visiting aims to achieve diverse outcomes in diverse contexts. The Precision Paradigm explicitly cites home visiting experience as well as theory and empirical evidence to reflect the field’s commitment to partnership across stakeholder groups and thus the central role of families and front-line providers in intervention design and testing. It does not call for the use of specific theories of behavior change or empirical evidence; rather it mandates the well-reasoned use of these in intervention design and research.

### Study purpose

One foundational aspect of this work is to characterize the interventions currently comprising evidence-based home visiting models in a granular, standardized and generic way. This was the broad goal of the project reported here, which focused specifically on interventions to promote good birth outcomes. The project identified models’ intended behavioral pathways to good birth outcomes and their stance on home visitors’ use of specific intervention techniques to promote families’ progress along intended pathways. It was intended as a pilot test of methods to define behavioral pathways and intervention techniques across other intended home visiting outcomes. These are some of the building blocks of a more explicit foundation for workforce development, service monitoring and cross-model research to understand what works best for whom.

We focused on home visiting interventions to promote good birth outcomes for three reasons. First, birth outcomes are highly variable across population subgroups and thus are a major public health concern. Second, this ‘test of concept’ project could be carried out more efficiently by collaborating with a small number of evidence-based models – the subset whose range of intended outcomes includes birth outcomes. Lastly, the project could draw on a solid body of research on major risks contributing to poor birth outcomes and maternal behaviors to reduce those risks.

## Methods

**Fig. 2:**
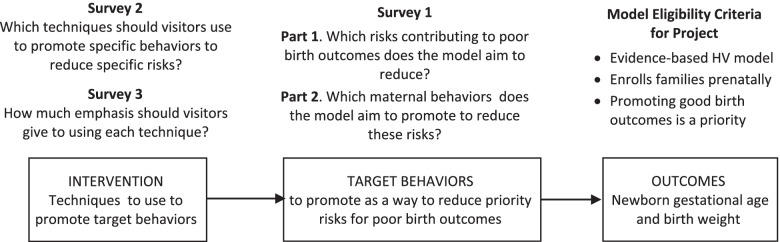
Home visiting model eligibility and data collection mapped to the home visiting Precision Paradigm

Co-authors affiliated with HARC’s coordinating center conceptualized and designed the observational study, gathered and analyzed data, interpreted study results, and drafted and revised this manuscript. The Strengthening the Reporting of Observational Studies in Epidemiology (STROBE) guidelines for reporting observational studies [22] was used to ensure rigorous reporting of the study (see additional information). National model leadership identified staff to take the lead as their representatives and co-authors for this study. Co-authors representing home visiting models provided study data, interpreted results, and revised the manuscript. Figure 2 illustrates how sample selection and data collection mapped to parts of the Precision Paradigm. As shown, reading from right to left in the figure, study eligibility was based on a model’s intended outcomes, Survey 1 focused on models’ target behaviors, and Surveys 2 and 3 focused on their expectations regarding intervention techniques.

### Selection of home visiting models

The intended sample was evidence-based models enrolling families prenatally to promote healthy birth outcomes. We identified models meeting three criteria: designated as evidence-based by the Home Visiting Evidence of Effectiveness (HomVEE) review; implemented in states or tribal communities in the United States; and enrolling families prenatally. Eight models met these criteria. We contacted each model’s national office to ascertain whether the model aimed to prevent premature birth or low birth weight, defined as a birth < 37 weeks gestation and a birth weight < 2500 g. Two of the eight models indicated that promoting healthy birth outcomes was not a central focus. The other six models – Family Spirit, Kentucky’s Health Access Nurturing Development Services (HANDS), Healthy Families America, Minding the Baby, Nurse-Family Partnership, and Parents as Teachers – indicated that improving birth outcomes was one of their intended outcomes. Five of these models agreed to participate in the project; state administrators for HANDS declined due to demands of the COVID-19 pandemic.

### Data collection

Model representatives completed three surveys mapped to the Precision Paradigm. The surveys worked backward, from ascertaining the risks the model aimed to reduce, to the maternal behaviors it aimed to promote to reduce those risks, to the techniques it endorsed visitors to use to promote those behaviors and the emphasis to give to each (Fig. 2). All surveys were developed for this study and have not been previously published elsewhere (see Additional files 1, 2 and 3).

HARC coordinating center investigators distributed each survey to model representatives at the same time, for independent completion within 2–3 weeks. Surveys 2 and 3 were distributed after all models had completed the preceding survey. HARC coordinating center investigators encouraged model representatives to ask for clarification if they were uncertain how to answer a question. Model representatives submitted seven questions. HARC investigators emailed responses to all five models by the next working day.

### Measurement

#### Survey 1—intended pathways

HARC investigators drew from the literature [23–27] and from relevant American College of Obstetrics and Gynecologists Committee Opinions [28] to identify modifiable risks for low birth weight and premature birth, and target behaviors to reduce these risks. These are the birth outcomes most often used in home visiting impact studies in the US. While infant mortality is a Sustainable Development Goals indicator [29], and the US ranks poorly for this indicator [30], we did not use it in this project because it has not been used in home visiting impact studies in the US and because prematurity is the second leading cause of infant mortality.

To minimize respondent burden, Part 1 of Survey 1 was limited to ten common, diverse, modifiable, evidence-based risk factors that could be reduced through home visiting and that fell within the scope of the current pregnancy. The risks fell into four groups: 1) health care use (inadequate prenatal care); 2) psychosocial well-being (high stress, depression, intimate partner violence); 3) behavioral health (tobacco use, alcohol use, illicit drug use); and 4) biologic risk factors (infection, diabetes, high blood pressure). The survey asked representatives to rate the priority their model gave to reducing each risk. Response choices were: *not a priority*, *low priority*, *moderate priority*, *high priority*, and *not sure*. A *priority risk* was defined as one whose reduction was designated as a low, moderate, or high priority.

Part 2 of the survey focused on 14 behaviors that could be promoted within home visiting for the current pregnancy. We saw these behaviors as falling into four groups: 1) basic health promotion (physical activity, healthy diet, stress reduction activities, social supports); 2) health care use (adherence to prenatal care visit schedule, engagement in substance use treatment, and alerting the prenatal care provider to warning signs; 3) behavioral health (stopping or reducing tobacco use, stopping or reducing alcohol use, stopping or reducing illicit drug use); and 4) specific risk reduction behaviors (condom use, developing a domestic violence safety plan, medication adherence, self-monitoring of physiologic indicators). The survey asked representatives to rate their models’ expectations of home visitors for promoting specific maternal behaviors to reduce each of its priority risks. Response choices were *required*, *recommended but not required*, *no expectation but compatible with our model*, *not compatible with our model*, and *not sure*. A *target behavior* was defined as a behavior the model either required or recommended visitors to promote.

The ten risks and 14 behaviors together defined 41 unique pathways to good birth outcomes (Table 1). The literature recommended some behaviors as a way reduce multiple risks. For example, physical activity is a behavior to reduce high stress, depression, high blood pressure and diabetes. Of note, the literature characterized three risk factors – tobacco use, alcohol use and inadequate prenatal care – not only as risk factors but as behaviors influencing other risk factors. In the same way, we defined these three constructs as both risk factors and maternal behaviors.

**Table 1:** Scope of survey 1: potential pathways to promote good birth outcomes^a^

	**Health Care Risk**	**Psychological Risks**			**Behavioral Health Risks**			**Biologic Risks**		
**Target Maternal Behaviors**	**Inadequate PNC**^**b**^	**High Stress**	**Depression**	**IPV**^**c**^	**Tobacco Use**	**Alcohol Use**	**Illicit SU**^**d**^	**Infection**^**e**^	**Diabetes**	**High Blood Pressure**
**Basic Health Promotion**										
Engage in physical activity		X	X						X	X
Adhere to a healthy diet									X	X
Engage in stress reduction activities		X	X	X	X	X	X			
Use social supports		X	X	X	X	X	X			
**Health Care Use**										
Adhere to PNC visit schedule	X		X						X	X
Engage in SU treatment							X			
**Behavioral Health**										
Stop or reduce tobacco use					X					X
Stop or reduce alcohol use						X				X
Stop or reduce illicit SU							X			
**Specific Risk Reduction Behaviors**										
Use condoms								X		
Develop a safety plan				X						
Alert PNC provider to warning signs			X						X	X
Adhere to medication regimen			X		X		X	X	X	X
Self-monitor physiologic indicators									X	X

^a^Each X represents a unique pathway to good birth outcomes by promoting a specific target behavior to reduce a specific risk contributing to poor birth outcomes. There are 41 pathways

^b^Inadequate prenatal care is defined as late entry or inadequate number of visits post enrollment in HV

^c^Intimate partner violence

^d^Substance use (heroin or cocaine)

^e^Sexually transmitted, vaginal, or urinary tract

At the end of Survey 1, HARC coordinating center investigators used each model’s priority risks and target behaviors to define its *intended pathways* to good birth outcomes. An *intended pathway* for a model is one linking a target behavior with a priority risk. Each model could have up to 41 intended pathways; the number and nature of intended pathways depended on the model’s priority risks and target behaviors to reduce those risks.

#### Survey 2—endorsement of intervention technique categories in intended pathways

Survey 2 asked respondents to rate their models’ stance regarding home visitors’ use of each of 23 technique categories for each of its intended pathways. Response choices were *required*, *recommended but not required*, *no expectation but compatible with our model*, *not compatible with our model*, and *not sure*. An *endorsed technique category* was defined as one that the model required or recommended visitors to use for a specific intended pathway.

The Appendix describes the 23 technique categories. HARC coordinating center investigators defined them by adapting an existing taxonomy of behavior change techniques and by adding techniques commonly used in home visiting but not represented in the existing taxonomy. The existing taxonomy contained 93 techniques grouped into 16 categories and was defined by applying consensus building methods to techniques identified in the behavior change literature [17]. We used technique categories rather than individual techniques to reduce respondent burden. We modified these categories in four ways: 1) split four of the original categories into eight narrower, more homogeneous categories; 2) dropped one of the original categories but assigned some of its techniques to another existing category; 3) added the category, “assess readiness for change,” because it is concordant with a family-centered approach and with theories of behavior change that differentiate motivating, enabling, and reinforcing target behaviors [31]; and 4) added three categories aligned with the framework of West et al. [32] to reflect home visiting’s use of referral and coordination.

#### Survey 3—emphasis in using endorsed technique categories

Survey 3 explored how much models expected home visitors to emphasize technique categories within selected intended pathways. To minimize respondent burden while maximizing the number of comparisons that could be made, the survey’s focus was limited to a subset of pathways defined by behaviors designated as target behaviors by all five models and associated with reducing multiple risks.

Within that subset of pathways, each model’s version of Survey 3 was also limited to the model’s intended pathways as determined by Survey 1 and the technique categories it had endorsed for those pathways in Survey 2. For each pathway-specific set of endorsed technique categories, the model’s representative rated the relative emphasis the model expected visitors to give to each technique category. Response choices were adapted from those of Smith et al. [33] and ranged from one (low emphasis) to five (high emphasis) and no stance. Response choices two through four were not labeled. A technique category with a rating of five was defined as a high-emphasis technique category.

### Analysis

HARC coordinating center investigators carried out data analyses. After all surveys had been completed, we shared results with model representatives in several iterations, using representatives’ feedback to guide new analyses and to improve the clarity and usefulness of results.

#### Priority risks, target behaviors and intended pathways

We described the distribution of model responses for each risk. We determined and graphed the number of models designating each of Table 1’s 41 behavioral pathways as an intended pathway.

#### Stance on technique categories

We calculated the percent distribution of each model’s responses (required, recommended, no expectation but compatible, not compatible, and not sure) for each technique category across all of its intended pathways combined. For all models combined, we calculated the mean of the model-specific percent distribution of responses.

#### Emphasis on technique categories

We calculated the number of models designating each technique category as a high-emphasis technique category in any of the intended pathways in their version of Survey 3. We elaborated on this for each of four pathways to reduce maternal depression through four target behaviors – physical activity, adherence to the prenatal care visit schedule, stress reduction and social support. For each of these pathways, we calculated the number of models endorsing each technique category at all and as a high-emphasis technique category.

#### Comparison of models’ priority risks, intended pathways and stance on technique categories

We determined each model’s number of priority risks, target behaviors, and intended pathways. We calculated the number of technique categories each model endorsed, required, recommended, rated as compatible while not specifically endorsed, and rated as not compatible on one or more of the model’s intended pathways. We measured each model’s propensity for explicit endorsement of technique categories by calculating the mean number of categories it endorsed per pathway across its intended pathways. We measured each model’s consistency in endorsing specific technique categories across pathways as the percent of its technique categories that it either always or never endorsed across all its intended pathways. We report the minimum, median and maximum value for all of these model-specific measures.

### Discussion and interpretation of results

HARC coordinating center investigators prepared results tables and talking points for three rounds of independent review and written feedback by model representatives followed by group discussion of the collated feedback.

## Results

### Participating home visiting models

The five models varied in enrollment eligibility criteria, educational requirements for hire as home visitors, and theories of change (Table 2). In general, years of dissemination was positively associated with number of local implementing agencies.

**Table 2:** Characteristics of participating home visiting models

	**Year Designated as EBHV**^**a**^	**Eligibility for Enrollment**	**Home Visitor Educational Requirements for Hire**	**Underlying Theories or Frameworks**	**Year Scale Up Began**	**Number of LIAs**^b^
Family Spirit	2015	Expectant women and families with children < 3 years old	Paraprofessional health educators with at least a high school credential	G.R. Patterson’s developmental model; Traditional Tribal Teachings	2006	54
Healthy Families America	2011	Expectant women and families with newborns. HFA sites determine additional eligibility criteria.	Home visitors with at least a high school diploma or bachelor’s degree, depending on state or agency needs	Attachment theory; Bio-ecological systems theories; Dyadic; Trauma-Informed Care; Strength Based/Adaptive	1992	574*
Minding the Baby	2014	First-time expectant women in their 2^nd^ or 3^rd^ trimester of pregnancy	Nurse or mental health professionals. Mental health visitors must have a master’s in social work or a related field	Attachment theory; Reflective parenting; Self-efficacy; Social-Ecological	2002	4*
Nurse-Family Partnership	2011	First-time expectant women in 1^st^ or 2^nd^ trimester of pregnancy with low-income. NFP sites determine additional eligibility criteria.	Nurses with at least a bachelor’s degree	Attachment theory; Human Ecology theory; Self-Efficacy; Social-Cognitive	1996	312*
Parents as Teachers	2011	Expectant women and families with children up to kindergarten entry (usually age 5)	Paraprofessional parent educators with at least a high school credential; bachelor’s degree in early child education recommended	Attribution theory; Developmental Parenting; Family Systems Theory; Human Ecology Theory	1985	987*

^a^Year designated as evidence-based by HomVEE [3]

^b^Number of local implementing agencies in the United States [34]

Models with an asterisk(*) have been disseminated internationally

### Models’ priority risks

Each risk was designated as a priority risk by at least four models (Table 3). All models made it a high priority to reduce high stress. All five prioritized reducing inadequate prenatal care, maternal depression, intimate partner violence, tobacco use and alcohol use, but some rated these as a moderate versus high priority. Four models rated reducing illicit substance use as a high priority; women known to use illicit substances were not eligible for enrolling in the other model and thus this was not a priority risk for that model. Each biologic risk was a priority risk for four models, though not the same four models. Biologic risks were not a high priority for most models.

**Table 3:** Cross-model^a^ distribution of responses regarding priority given to reducing specific risks

Risk Category	Specific Risk	High Priority	Moderate Priority	Low Priority	Not a Priority
Health care risk	Inadequate prenatal care	4	1		
Psychosocial risks	High stress	5			
	Depression	4	1		
	Intimate partner violence	3	2		
Behavioral health risks	Tobacco use	3	2		
	Alcohol use	4	1		
	Illicit substance use	4			1
Biologic risks	Infection	2	1	1	1
	Diabetes	2	2		1
	High blood pressure	1	2	1	1

^a^*N* = 5 models

### Models’ intended pathways to good birth outcomes

**Fig. 3:**
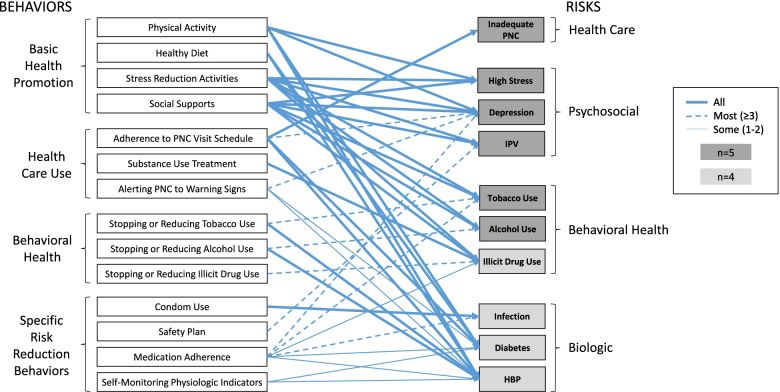
Models’ intended pathways from target maternal behaviors to priority risks

Each of the 41 behavioral pathways was an intended pathway for at least one model (Fig. 3). Pathways involving basic health promotion behaviors were always designated as intended pathways. Adhering to the prenatal care visit schedule was more often a part of intended pathways than was alerting the prenatal care provider to observed warning signs.

Tobacco and alcohol use reduction were more likely to be designated on intended pathways if the intent was to reduce the risk of high blood pressure rather than the risks of these behaviors themselves. Condom use was part of an intended pathway for all models that designated infection as a priority risk. Models were less likely to designate safety plans, medication adherence and self-monitoring of physiologic indicators as part of intended pathways.

### Models’ stance on technique categories

Table 4 summarizes models’ stance regarding use of technique categories. Across all models combined, 11 technique categories were endorsed in ≥ 75% of intended pathways and eight were endorsed in 50–74% of intended pathways. Thus, nearly all technique categories were endorsed in at least half of intended pathways.

**Table 4:** Cross-model distribution of expectations for visitors’ use of specific technique categories in intended pathways^a^ to good birth outcomes

**How Often Technique Category is Endorsed**^b^	**Technique Number and Name**^c^		**Required**	**Recommended**	**No Expectation but Compatible**	**Not Compatible**	**Models Designating as High-Emphasis**^**d**^
≥ 75% of intended pathways	T20	Self-Belief	42%	42%	16%	0%	3
	T03	Monitoring and Feedback	37%	48%	15%	0%	3
	T22	Monitoring and Follow-up of Referral	35%	56%	9%	0%	3
	T21	Referral and Linkage	32%	59%	9%	0%	4
	T01	Assess Readiness for Change	30%	49%	21%	0%	1
	T14	Credible Source	27%	63%	10%	0%	3
	T23	Coordination with Other Services	18%	73%	9%	0%	3
	T02	Goals and Planning	16%	73%	11%	0%	3
	T04	Provide Social Support	15%	72%	13%	0%	3
	T05	Suggest or Arrange Social Support^e^	13%	73%	15%	0%	2
	T08	Antecedents	8%	75%	17%	0%	1
50–74% of intended pathways	T06	Natural Consequences	10%	51%	39%	0%	1
	T12	Repetition and Substitution^e^	8%	60%	33%	0%	0
	T13	Comparison of Outcomes^e^	8%	58%	35%	0%	2
	T07	Shape Knowledge of Behavior	8%	54%	38%	0%	1
	T11	Associations to Deter Unwanted Behavior	8%	51%	41%	0%	0
	T10	Associations to Promote Wanted Behavior	8%	50%	42%	0%	0
	T17	Mental Regulation	4%	63%	33%	0%	2
	T09	Behavior Observation	1%	63%	35%	0%	1
25–49% of intended pathways	T15	Incentives and Rewards	8%	35%	57%	0%	0
	T19	Self-Identity	3%	32%	39%	26%	1
< 25% of intended pathways	T18	Identity as Example to Others	6%	7%	87%	0%	0
	T16	Scheduled Consequences	0%	0%	1%	99%	0

^a^An *intended pathway* is one in which a model designates a maternal behavior as a *target behavior* by requiring or recommending that visitors promote it as a way to reduce a *priority risk*

^b^A technique category is considered endorsed if the model either requires or recommends that the visitor use it in the context of an intended pathway

^c^Number and name as in the Appendix

^d^A model was considered to designate the technique category as high-emphasis if it rated it a “5” for any of its intended pathways in Survey 3

^e^Percentages do not total 100% due to rounding

Model representatives judged only two technique categories as not compatible with their model. One of these technique categories was self-identity; models’ stance on this varied enormously. Some models required use of this technique on some pathways while others designated it as not compatible in all instances (not shown in table). The other category, scheduled consequences, was virtually always defined as not compatible with the model.

### Models’ stance on the relative emphasis to give technique categories

One model never designated any technique category as high-emphasis for any pathway. The other four models designated some technique categories as high-emphasis for at least some pathways. All four models designated referral and linkage as a high-emphasis technique category (Table 4, last column). Seven other technique categories were designated as a high-emphasis technique category by three models and nine were so designated by one or two models.

**Fig. 4:**
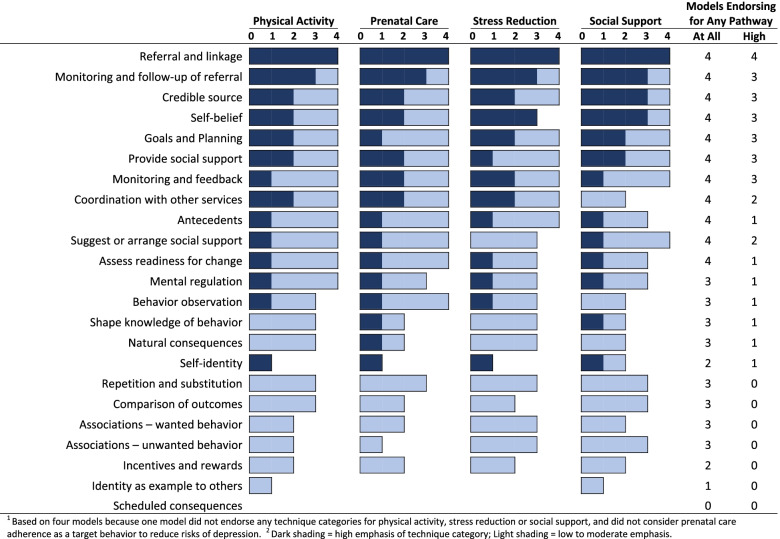
Models^1^ endorsing and emphasizing technique categories^2^ to promote four target behaviors to reduce maternal depression

Figure 4 illustrates more detailed results for four pathways to reduce maternal depression. The number of models endorsing a technique category is similar across the four pathways. The number of models designating a technique category as high-emphasis is also similar across the four pathways. However, within pathways, models often differed in their stance on particular technique categories. For example self-identity was designated as a high-emphasis technique category by one model, but rarely endorsed at all by other models.

### Cross-model similarities and differences

The five models varied considerably on their priority risks, target behaviors, intended pathways and stance on technique categories (Table 5). For example, the number of behaviors they designated as target behaviors ranged from five to 14, and their number of intended behavioral pathways ranged from 16 to 41.

**Table 5:** Models’ range in priority risks, target behaviors, intended pathways and stance on technique categories

	**Across the Five Models**		
	**Minimum**	**Median**	**Maximum**
**Priority Risks, Target Behaviors and Intended Pathways**			
Number of Priority Risks	7	10	10
Number of Target Behaviors	5	12	14
Number of Intended Pathways	16	34	41
**Stance on the 23 Technique Categories**			
Number ever Endorsed	12	20	23
*Number ever Required*	*0*	*5*	*22*
*Number ever Recommended*	*12*	*18*	*20*
Number ever Not Endorsed but Compatible with Model	2	15	23
Number ever Not Compatible	1	1	2
**Propensity toward Explicit Endorsement of Technique Categories**			
Mean Number of Endorsed Technique Categories per Intended Pathway	1.5	16.0	20.0
**Consistency of Technique Category Endorsement**			
Percent of Technique Categories that are Either Always or Never Endorsed across All Intended Pathways	22%	70%	100%
**Breadth of Designated High-Emphasis Technique Categories**			
Number of Technique Categories ever Defined as High-Emphasis	0	10	12

The models varied considerably in their stance on home visitors’ use of specific behavior change technique categories. All models required or recommended use of some technique categories, but they varied from endorsing as few as 12 to as many as all 23 technique categories. At least one model never required use of any technique category, while another required use of 22 of the 23 technique categories for at least some intended pathways. The models were similar in one respect; they all designated only one or two technique categories as not compatible with their model.

The models also varied greatly in how often they explicitly endorsed technique categories to use on intended pathways, ranging in this from 1.5 to 20 endorsed technique categories per intended pathway. The models differed greatly in their consistency in endorsing technique categories across intended pathways; the percent of technique categories a model consistently endorsed or not endorsed ranged from 22–100%.

## Discussion

Home visiting is building a new research paradigm to achieve greater precision. Reasons include the legislative mandate to individualize services [1]; empirical evidence that family engagement has remained challenging [5] and that average effects have remained small over many years [6, 7]; and the conclusion of systematic reviews and meta-analytic research that past research methods and reporting practices seriously compromise the ability to identify core components [8]. A shift toward precision also aligns with shifts toward precision in health care [35] and public health [36] in general.

The new research paradigm requires not only innovative study designs [37] but also a common over-arching conceptual framework and language to support shared learning. Lack of a common framework and language yield unclear, inconsistent descriptions of interventions. This frustrates what can be learned from systematic reviews and meta-analytic studies. This is true for home visiting [8, 38] and also for interventions that can be implemented in a range of settings, for example to promote behavioral health [17] and positive parenting behavior [39]. Even more important for home visiting’s evolution, lack of a common over-arching framework and language hampers stakeholders’ collaboration and co-learning in developing clear, coherent, effective interventions and in understanding the similarities and differences of existing evidence-based models.

Evidence-based models play a critical role in shifting the research paradigm because they are part of the context for defining and testing existing and emerging interventions. This study confirmed the feasibility of using a standardized approach to elicit models’ intended behavioral pathways and their stance on techniques to support families’ progress on those pathways. To our knowledge, this is the first home visiting study to use this approach. Results have implications for future research, policy and practice to promote precision in home visiting.

Models identified many intended pathways and differed in their sets of such pathways. This complexity and variation speak to the need for valid and reliable tools to assess risks and target behaviors, and the potential value of prioritizing intended pathways. Those who design new interventions for use within existing home visiting models must be aware of the fit of new interventions with models’ existing priority risks and target behaviors. Emerging research funding opportunities for innovation toward precision home visiting make clear the growing need for a common framework and language not only to specify innovations, but also to define and differentiate the context of the home visiting models in which innovations will be implemented [40, 41].

While models differed in their mix of intended pathways, it is premature to suggest whether and how to triage families across models based on this aspect of model differentiation. Many communities offer only one model, and triage policies must consider many factors beyond models’ intended pathways.

Models ranged from 1.5 to 20.0 in the average number of technique categories they endorsed per intended pathway. This variation might reflect philosophical differences across models on prescribing the techniques that visitors are to use versus delegating prescription of techniques to implementing agencies versus not conceptualizing services in terms of techniques to promote target behaviors. For models that delegate, research is needed to learn how implementing agencies decide which techniques to endorse. For all models, it is important to ascertain how clearly model expectations are communicated to implementing agencies and how adequately implementation systems support staff to use expected techniques. An upcoming report from the current project focuses on this issue.

Model representatives rated nearly all behavior change technique categories as compatible with their models. This suggests that intervention developers could draw from a broad portfolio of techniques in designing interventions compatible with existing evidence-based models.

Models varied in the consistency of their expectations for using and emphasizing technique categories across intended pathways. Some models had consistent expectations across all pathways. For such models, results for one pathway might be generalizable to other pathways. But a model’s varied expectations across pathways raises interest in how differences in expectations are conveyed to front line staff via implementation systems.

### Limitations

Several study limitations should be addressed in future work. The project considered only prenatal home visiting to promote good birth outcomes and focused only on two components of the Paradigm – target behaviors as a part of intended pathways, and intervention techniques. Parallel work is needed for home visiting’s other intended outcomes. For all outcomes, complementary work is needed to identify models’ underlying theories of behavior change, expected links from techniques to mechanisms of action, and how such links might be moderated by context. Many of the underlying theories of home visiting are explanatory theories rather than theories of behavior change; future work needs to specify underlying theory in terms of mechanisms of action and mediating target behaviors.

The project’s operationalization of intervention techniques was limited in several ways. We used technique categories rather than individual techniques to reduce respondent burden; future work should investigate the value of focusing on individual techniques. We drew from a highly regarded taxonomy of behavior change techniques but had to add several categories for techniques commonly used in home visiting such as referral, coordination, and assessing readiness for change. Rigorous developmental work is underway to refine these categories. While our list of technique categories can be applied to interventions across a broad range of outcomes, its completeness and appropriateness – as judged by varied home visiting stakeholders – have yet to be confirmed. This, too, is the subject of ongoing work to build the Paradigm.

The project focused much more on content-based than relational techniques. Content-based techniques reflect *what* is delivered while relational techniques reflect *how* it is delivered. Home visiting has always valued the importance of a strong working relationship between family and home visitor. This argues for incorporating relational techniques such as those of motivational interviewing [15] and of communication strategies to achieve the social-emotional and activation functions of social interaction [16, 42–45]. In debriefing, models considered the distinction between techniques to establish a working relationship and the relationship itself. They agreed on the critical need for research to confirm the influence of relational links with the building of a strong working relationship, and to test the working relationship itself as a mediator of behavior change.

In debriefing, model representatives also considered methodologic issues regarding the designation of target behaviors and technique categories as “required” versus “recommended”. They shared the process each had used to determine their model’s stance. They discussed their models’ mechanisms to communicate expectations to local implementing agencies, for example through training, curricula and the credentialing process. They discussed implications of the distinction between a requirement and a recommendation for implementation systems and local program operations. Discussion revealed the need to distinguish model expectations per se from mechanisms to convey expectations to local programs and to support staff to meet them.

Every part of the Precision Paradigm is important. This project’s focus on target behaviors and intervention techniques is not to imply that these are the most salient parts of the Paradigm. Rather, we focused on these because we believed it was important to test usability of the Paradigm early on and we felt that these two parts would be easiest for home visiting models to define.

Our approach was complex and needs to be streamlined. An example was our use of priority risks and target behaviors to define intended behavioral pathways. Future work should explore the feasibility of considering target behaviors alone. Whether using the term ‘core components’, ‘active ingredients’ or ‘common features’, it is ambitious to build a common framework and language for granularity in a field that has traditionally focused on full models. There are, no doubt, many ways to make our approach more parsimonious and the language of the Paradigm clearer and more concise. This is the shared task of home visiting stakeholders, as described below in Future Work.

### Practical applications

This project assessed whether national models could use the Paradigm to define intended behavioral pathways and expectations for visitors’ use of specific behavior change technique categories to promote good birth outcomes. Our intent was to pave the way for much broader stakeholder engagement in using the Paradigm to advance the field. To that end, HARC brings stakeholders together to specify each component of the Precision Paradigm, to develop and apply research methods using it, and to address high priority research issues through shared learning grounded in actionable empirical research. Such issues include:

- The coherence of interventions as indicated by defining intervention techniques, mechanisms of action, and target behaviors in alignment with family assets, needs, interests and concerns in pursuing intended outcomes.
- The clarity of interventions as indicated by the concordance of home visitors’ perceptions with models’ and implementing agencies’ expectations regarding intended pathways and intervention techniques.
- The adequacy of implementation systems to motivate, enable and reinforce home visitors’ competent use of intervention techniques.
- Identification of intervention techniques in models using more of a psychosocial than a behavioral approach to achieving intended outcomes.
- How techniques are combined into interventions, and how relational and content techniques work together to promote behavior change.
- The influence of interventions on presumed mechanisms of action for target behaviors and how context moderates this. Prior research suggests that evidence of the links between techniques and mechanisms of action is far from definitive [46].
- How family and community context moderates the acceptability and effectiveness of specific techniques and interventions, and the resulting impact of interventions in achieving health equity.

### Future work

The work reported here demonstrated the feasibility of defining models’ intended pathways and stance on behavior change technique categories. Two complementary parts of the project are now nearing completion. The first of these, a survey of local programs, assesses local programs’ perspectives on intended pathways and technique categories and the strength of current implementation systems to support their home visitors’ effective use of these technique categories. The second aspect, qualitative interviews with expectant families enrolled in home visiting, elicits their perspectives on the behavior change techniques used by their home visitors. The third, a review of the literature, assesses the completeness of reporting of components of the Paradigm in peer-reviewed reports of experimental testing of home visiting impacts on birth outcomes.

While this project applied the Paradigm to interventions targeted to enrolled families, it can be applied as well to implementation systems. First, use of the Paradigm in research to understand expectations of home visitors is foundational for designing implementation systems to support home visitors to meet those expectations. Second, the Paradigm can be used to assess the adequacy of implementation systems, by conceptualizing expectations of home visitor practice behaviors as the “target behaviors” and implementation system components as the interventions. In related work, HARC is developing resources to support the field in using the Paradigm in implementation research.

The models that participated in this project are exemplars for other stakeholders whose perspectives are also critical in innovation toward precision home visiting. HARC’s coordinating center is working with diverse groups to build the Precision Paradigm and promote its use in innovative research toward precision home visiting. Part of this involves identifying theories of behavior change that are currently used, or might be used, to specify interventions that could be implemented in home visiting. Our methods include qualitative approaches to complement the quantitative approach used in this project. We are working not only with evidence-based models, but with representatives of promising programs, public agencies and home visiting funders, and with workforce developers and implementation researchers. We are mining the wisdom and culture of the families who enroll in home visiting and the home visitors who provide services, as reflected in the paradigm’s reliance on home visiting experience to complement theory and empirical evidence.

To accelerate this work, HARC makes the resources it develops publicly available via its website [19] technical assistance to stakeholder teams, and Open Science publication. The goal is to build all stakeholders’ capacity for innovative precision home visiting research to advance the field.

## Conclusions

Evidence-based home visiting models successfully used the Paradigm to articulate in a standardized way their intended behavioral pathways to good birth outcomes and their expectations for home visitors’ use of behavior change techniques to promote families’ progress on these pathways. The Precision Paradigm is a promising resource to accelerate innovative cross-model research to clarify which interventions within home visiting work best for which families, in which contexts, why and how.

### Supplementary Information

**Additional file 1.**

**Additional file 2.**

**Additional file 3.**

## Data Availability

The de-identifiable data that support the findings of this study are available from the corresponding author upon reasonable request through an institutional data sharing agreement that includes written permission of the home visiting models that participated in the study.

## Appendix

Technique categories and operational definitions.

**Table:**

1	**Assess Readiness for Change** Gather information about the expectant woman’s readiness to change the behavior.
2	**Goals and Planning** Assist the expectant woman to: set a behavior change goal; develop a plan to meet the goal using strategies to overcome barriers and increase facilitators; review her progress toward the goal; modify the goal or plan as needed.
3	**Monitoring and Feedback** Monitor the expectant woman’s progress in changing the behavior; give feedback on that progress; establish ways for the expectant woman to self-monitor her progress.
4	**Provide Social Support** Directly provide the expectant woman encouragement, emotional support or practical help to perform the behavior.
5	**Suggest or Arrange Social Support** Suggest or assist the expectant woman to seek encouragement, emotional support or practical help to perform the behavior from a friend, relative, colleague, or group.
6	**Natural Consequences** Provide written, verbal or visual information about the behavior’s health, emotional, social or environmental consequences; encourage her to assess her feelings after attempts to perform the behavior; raise her awareness of future regret about performing the unwanted behavior.
7	**Shape Knowledge of Behavior** Provide information or instruction to shape the expectant woman’s knowledge of *how to perform the behavior*. This includes identification of behavioral ‘triggers’ and their perceived causes. ‘Triggers’ are thoughts or situations that lead to performance of the unwanted behavior.
8	**Antecedents** Change or support change of the expectant woman’s physical or social surroundings to facilitate performing the behavior, create barriers to an unwanted behavior, or avoid cues to an unwanted behavior.
9	**Behavior Observation** Demonstrate the behavior; provide an observable example of the behavior; draw attention to others’ performance of the behavior as a model.
10	**Associations to Promote Wanted Behavior** Identify, introduce, or alter social or environmental prompts or cues to promote the wanted behavior.
11	**Associations to Deter Unwanted Behavior** Identify, alter, or remove social or environmental prompts or cues to deter the unwanted behavior.
12	**Repetition and Substitution** Encourage the expectant woman to practice performing the behavior or substitute it for an unwanted behavior.
13	**Comparison of Outcomes** Encourage the expectant woman to compare the pros and cons of changing the behavior, or to compare the outcomes of changing versus not changing the behavior. Includes encouraging the expectant woman’s imagination or observation of either the consequences of the unwanted behavior or rewards for the wanted behavior.
14	**Credible Source** Present verbal or visual communication from a credible source in favor of or against the behavior.
15	**Incentives and Rewards** Provide or arrange for the expectant woman to receive a *material incentive or reward* (something of value) or a *social incentive or reward* (words of congratulation), or removal of an unpleasant consequence for making progress in performing the behavior. Includes encouraging the expectant woman to use self-incentives or self-rewards.
16	**Scheduled Consequences** Use a threat of future punishment or removal of a reward as a consequence of performance of an unwanted behavior; arrange for a negative consequence or punishment following performance of an unwanted behavior.
17	**Mental Regulation** Suggest strategies to minimize demands on the expectant woman’s mental resources to make it easier for her to perform the behavior.
18	**Identity as Example to Others** Suggest to the expectant woman that performing the behavior might serve as an example to others.
19	**Self-identity** Assist the expectant woman to identify discrepancies between her behavior and her values or self-image; encourage her to self-identify as someone who *used to* perform the unwanted behavior; suggest her adopting a new perspective to change thoughts or emotions about the behavior.
20	**Self-belief** Promote the expectant woman’s self-belief that she can successfully perform the behavior, for example by persuading her about her capabilities and encouraging her to mentally rehearse success, focus on past success or use positive self-talk.
21	**Referral and Linkage** Provide referral or information to link the expectant woman to a community resource to assist in performing the behavior; review progress in completing the referral; support connections in completing the referral or perform an interagency case review.
22	**Monitoring and Follow-up of Referral** Review the expectant woman’s experience accessing community resources to help her perform the behavior; assist in overcoming barriers to completing a referral.
23	**Coordination with Other Services** Ask about and act on the expectant woman’s ideas on how to assist her in adhering to guidance from other providers regarding performing the behavior.

## Abbreviations

EBHV: Evidence-based home visiting
HARC: Home Visiting Applied Research Collaborative
HFA: Healthy Families America
HomVEE: Home Visiting Evidence of Effectiveness
IPV: Intimate partner violence
LIA: Local implementing agency
MIECHV Program: Maternal, Infant and Early Childhood Home Visiting Program
NFP: Nurse-Family Partnership
PNC: Prenatal care
SU: Substance use
