# Supplementary material for: Changing the home visiting research paradigm: models’ perspectives on behavioral pathways and intervention techniques to promote good birth outcomes
Source: BMC Public Health. 2022 May 21;22:1024. doi: 10.1186/s12889-022-13010-5 (PMC9123293; doi:10.1186/s12889-022-13010-5)
Supplement: Supplementary file 1 — Additional file 1. [file 12889_2022_13010_MOESM1_ESM.docx]

**Cross-Model Precision Prenatal HV Project Model Survey 1**

**Overview**

Thank you for taking part in the Cross-Model Precision Prenatal HV Project. The project objective is to describe techniques endorsed by evidence-based home visiting models to promote two birth outcomes, full-term birth and normal birth weight.

The purpose of this survey is to understand your model’s priorities for reducing each of 10 risk factors for poor birth outcomes and its expectations of home visitors for promoting each of 15 healthy behaviors to reduce one or more risk factors.

1. What is your name? (Text box) ____________________________________________
2. Please indicate the model you are representing.

- Family Spirit
- HFA
- Minding the Baby
- NFP
- PAT

3. In your model, how high a priority is it to address each of the following risk factors to promote a good birth outcome (full term birth or normal birth weight) for expectant women?

|  | Not a priority | Low priority | Moderate priority | High priority | Not sure |
| --- | --- | --- | --- | --- | --- |
| High blood pressure | 🞎 | 🞎 | 🞎 | 🞎 | 🞎 |
| Diabetes | 🞎 | 🞎 | 🞎 | 🞎 | 🞎 |
| Infection – sexually transmitted, vaginal, or urinary tract | 🞎 | 🞎 | 🞎 | 🞎 | 🞎 |
| Intimate partner violence | 🞎 | 🞎 | 🞎 | 🞎 | 🞎 |
| High stress | 🞎 | 🞎 | 🞎 | 🞎 | 🞎 |
| Depression | 🞎 | 🞎 | 🞎 | 🞎 | 🞎 |
| Tobacco use | 🞎 | 🞎 | 🞎 | 🞎 | 🞎 |
| Alcohol use | 🞎 | 🞎 | 🞎 | 🞎 | 🞎 |
| Illicit drug use – heroin or cocaine | 🞎 | 🞎 | 🞎 | 🞎 | 🞎 |
| Inadequate prenatal care – late entry or inadequate number of visits post enrollment in HV | 🞎 | 🞎 | 🞎 | 🞎 | 🞎 |

1. [For each question below, answer the question only if the response for the corresponding risk factor in Q3 is low, moderate, or high priority.]
2. What is your model’s expectation of home visitors for promoting each behavior below to reduce the risk of poor birth outcomes (low birthweight or preterm birth) **for expectant women with high blood pressure**?

Note: Check ‘Required’ if you expect home visitors to promote a behavior with ANY subset of expectant women with high blood pressure. You do not have to expect visitors to promote it with all expectant women with high blood pressure to check ‘Required’.

|  | Required | Recommended, but not required | No expectation, but compatible with our model | Not compatible with our model | Not sure |
| --- | --- | --- | --- | --- | --- |
| Engage in physical activity | 🞎 | 🞎 | 🞎 | 🞎 | 🞎 |
| Adhere to a healthy diet | 🞎 | 🞎 | 🞎 | 🞎 | 🞎 |
| Achieve a healthy gestational weight gain | 🞎 | 🞎 | 🞎 | 🞎 | 🞎 |
| Stop or reduce tobacco use | 🞎 | 🞎 | 🞎 | 🞎 | 🞎 |
| Stop or reduce alcohol use | 🞎 | 🞎 | 🞎 | 🞎 | 🞎 |
| Adhere to prescribed medication regimen | 🞎 | 🞎 | 🞎 | 🞎 | 🞎 |
| Self-monitor blood pressure | 🞎 | 🞎 | 🞎 | 🞎 | 🞎 |
| Adhere to prenatal care visit schedule | 🞎 | 🞎 | 🞎 | 🞎 | 🞎 |
| Alert prenatal care provider to warning signs of uncontrolled blood pressure | 🞎 | 🞎 | 🞎 | 🞎 | 🞎 |

>> NEXT PAGE >>

1. What is your model’s expectation of home visitors for promoting each behavior below to reduce the risk of poor birth outcomes (low birthweight or preterm birth) **for expectant women with** **diabetes**?

Note: Check ‘Required’ if you expect home visitors to promote a behavior with ANY subset of expectant women with diabetes. You do not have to expect visitors to promote it with all expectant women with diabetes to check ‘Required’.

|  | Required | Recommended, but not required | No expectation, but compatible with our model | Not compatible with our model | Not sure |
| --- | --- | --- | --- | --- | --- |
| Engage in physical activity | 🞎 | 🞎 | 🞎 | 🞎 | 🞎 |
| Adhere to a healthy diet | 🞎 | 🞎 | 🞎 | 🞎 | 🞎 |
| Achieve a healthy gestational weight gain | 🞎 | 🞎 | 🞎 | 🞎 | 🞎 |
| Adhere to prescribed medication regimen | 🞎 | 🞎 | 🞎 | 🞎 | 🞎 |
| Self-monitor blood glucose | 🞎 | 🞎 | 🞎 | 🞎 | 🞎 |
| Adhere to prenatal care visit schedule | 🞎 | 🞎 | 🞎 | 🞎 | 🞎 |
| Alert prenatal care provider to warning signs of uncontrolled diabetes | 🞎 | 🞎 | 🞎 | 🞎 | 🞎 |

1. What is your model’s expectation of home visitors for promoting each behavior below to reduce the risk of poor birth outcomes (low birthweight or preterm birth) **for expectant women at risk for infection (sexually transmitted, vaginal, or urinary tract)**?

Note: Check ‘Required’ if you expect home visitors to promote a behavior with ANY subset of expectant women at risk for infection. You do not have to expect visitors to promote it with all expectant women at risk for infection to check ‘Required’.

|  | Required | Recommended, but not required | No expectation, but compatible with our model | Not compatible with our model | Not sure |
| --- | --- | --- | --- | --- | --- |
| Use condoms | 🞎 | 🞎 | 🞎 | 🞎 | 🞎 |
| Adhere to prescribed medication regimen | 🞎 | 🞎 | 🞎 | 🞎 | 🞎 |

1. What is your model’s expectation of home visitors for promoting each behavior below to reduce the risk of poor birth outcomes (low birthweight or preterm birth) **for expectant women experiencing intimate partner violence**?

Note: Check ‘Required’ if you expect home visitors to promote a behavior with ANY subset of expectant women experiencing intimate partner violence. You do not have to expect visitors to promote it with all expectant women experiencing intimate partner violence to check ‘Required’.

|  | Required | Recommended, but not required | No expectation, but compatible with our model | Not compatible with our model | Not sure |
| --- | --- | --- | --- | --- | --- |
| Engage in stress reduction activities (such as meditation, mindfulness, breathing exercises, journaling, and performing an enjoyed activity) | 🞎 | 🞎 | 🞎 | 🞎 | 🞎 |
| Use social supports (such as seeking support from colleagues, friends, family, or support groups) | 🞎 | 🞎 | 🞎 | 🞎 | 🞎 |
| Develop a safety plan | 🞎 | 🞎 | 🞎 | 🞎 | 🞎 |

>> NEXT PAGE >>

1. What is your model’s expectation of home visitors for promoting each behavior below to reduce the risk of poor birth outcomes (low birthweight or preterm birth) **for expectant women with** **high stress**?

Note: Check ‘Required’ if you expect home visitors to promote a behavior with ANY subset of expectant women with high stress. You do not have to expect visitors to promote it with all expectant women with high stress to check ‘Required’.

|  | Required | Recommended, but not required | No expectation, but compatible with our model | Not compatible with our model | Not sure |
| --- | --- | --- | --- | --- | --- |
| Engage in stress reduction activities (such as meditation, mindfulness, breathing exercises, journaling, and performing an enjoyed activity) | 🞎 | 🞎 | 🞎 | 🞎 | 🞎 |
| Engage in physical activity | 🞎 | 🞎 | 🞎 | 🞎 | 🞎 |
| Use social supports (such as seeking support from colleagues, friends, family, or support groups) | 🞎 | 🞎 | 🞎 | 🞎 | 🞎 |

>> NEXT PAGE >>

1. What is your model’s expectation of home visitors for promoting each behavior below to reduce the risk of poor birth outcomes (low birthweight or preterm birth) **for expectant women with** **depression**?

Note: Check ‘Required’ if you expect home visitors to promote a behavior with ANY subset of expectant women with depression. You do not have to expect visitors to promote it with all expectant women with depression to check ‘Required’.

|  | Required | Recommended, but not required | No expectation, but compatible with our model | Not compatible with our model | Not sure |
| --- | --- | --- | --- | --- | --- |
| Engage in stress reduction activities (such as meditation, mindfulness, breathing exercises, journaling, and performing an enjoyed activity) | 🞎 | 🞎 | 🞎 | 🞎 | 🞎 |
| Engage in physical activity | 🞎 | 🞎 | 🞎 | 🞎 | 🞎 |
| Use social supports (such as seeking support from colleagues, friends, family, or support groups) | 🞎 | 🞎 | 🞎 | 🞎 | 🞎 |
| Adhere to prescribed medication regimen | 🞎 | 🞎 | 🞎 | 🞎 | 🞎 |
| Adhere to prenatal care visit schedule | 🞎 | 🞎 | 🞎 | 🞎 | 🞎 |
| Alert prenatal care provider to warning signs of harm to self or others | 🞎 | 🞎 | 🞎 | 🞎 | 🞎 |

>> NEXT PAGE >>

1. What is your model’s expectation of home visitors for promoting each behavior below to reduce the risk of poor birth outcomes (low birthweight or preterm birth) **for expectant women who use tobacco**?

Note: Check ‘Required’ if you expect home visitors to promote a behavior with ANY subset of expectant women who use tobacco. You do not have to expect visitors to promote it with all expectant women who use tobacco to check ‘Required’.

|  | Required | Recommended, but not required | No expectation, but compatible with our model | Not compatible with our model | Not sure |
| --- | --- | --- | --- | --- | --- |
| Engage in stress reduction activities (such as meditation, mindfulness, breathing exercises, journaling, and performing an enjoyed activity) | 🞎 | 🞎 | 🞎 | 🞎 | 🞎 |
| Adhere to prescribed medication regimen (such as smoking cessation aids) | 🞎 | 🞎 | 🞎 | 🞎 | 🞎 |
| Use social supports (such as seeking support from colleagues, friends, family, or support groups) | 🞎 | 🞎 | 🞎 | 🞎 | 🞎 |
| Stop or reduce tobacco use | 🞎 | 🞎 | 🞎 | 🞎 | 🞎 |

>> NEXT PAGE >>

1. What is your model’s expectation of home visitors for promoting each behavior below to reduce the risk of poor birth outcomes (low birthweight or preterm birth) **for expectant women who use alcohol**?

Note: Check ‘Required’ if you expect home visitors to promote a behavior with ANY subset of expectant women who use alcohol. You do not have to expect visitors to promote it with all expectant women who use alcohol to check ‘Required’.

|  | Required | Recommended, but not required | No expectation, but compatible with our model | Not compatible with our model | Not sure |
| --- | --- | --- | --- | --- | --- |
| Engage in stress reduction activities (such as meditation, mindfulness, breathing exercises, journaling, and performing an enjoyed activity) | 🞎 | 🞎 | 🞎 | 🞎 | 🞎 |
| Use social supports (such as seeking support from colleagues, friends, family, or support groups) | 🞎 | 🞎 | 🞎 | 🞎 | 🞎 |
| Stop or reduce alcohol use | 🞎 | 🞎 | 🞎 | 🞎 | 🞎 |

>> NEXT PAGE >>

1. What is your model’s expectation of home visitors for promoting each behavior below to reduce the risk of poor birth outcomes (low birthweight or preterm birth) **for expectant women who use illicit drugs (heroin or cocaine)**?

Note: Check ‘Required’ if you expect home visitors to promote a behavior with ANY subset of expectant women who use illicit drugs. You do not have to expect visitors to promote it with all expectant women who use illicit drugs to check ‘Required’.

|  | Required | Recommended, but not required | No expectation, but compatible with our model | Not compatible with our model | Not sure |
| --- | --- | --- | --- | --- | --- |
| Engage in stress reduction activities (such as meditation, mindfulness, breathing exercises, journaling, and performing an enjoyed activity) | 🞎 | 🞎 | 🞎 | 🞎 | 🞎 |
| Adhere to prescribed medication regimen | 🞎 | 🞎 | 🞎 | 🞎 | 🞎 |
| Use social supports (such as seeking support from colleagues, friends, family, or support groups) | 🞎 | 🞎 | 🞎 | 🞎 | 🞎 |
| Stop or reduce illicit drug use | 🞎 | 🞎 | 🞎 | 🞎 | 🞎 |
| Engage in substance use treatment | 🞎 | 🞎 | 🞎 | 🞎 | 🞎 |

1. What is your model’s expectation of home visitors for promoting the behavior below to reduce the risk of poor birth outcomes (low birthweight or preterm birth) **for expectant women with** **inadequate prenatal care (late entry or inadequate number of visits post enrollment in HV)**?

Note: Check ‘Required’ if you expect home visitors to promote a behavior with ANY subset of expectant women with inadequate prenatal care. You do not have to expect visitors to promote it with all expectant women with inadequate prenatal care to check ‘Required’.

|  | Required | Recommended, but not required | No expectation, but compatible with our model | Not compatible with our model | Not sure |
| --- | --- | --- | --- | --- | --- |
| Adhere to prenatal care visit schedule | 🞎 | 🞎 | 🞎 | 🞎 | 🞎 |

END OF SURVEY
