# Supplementary material for: Changing the home visiting research paradigm: models’ perspectives on behavioral pathways and intervention techniques to promote good birth outcomes
Source: BMC Public Health. 2022 May 21;22:1024. doi: 10.1186/s12889-022-13010-5 (PMC9123293; doi:10.1186/s12889-022-13010-5)
Supplement: Supplementary file 2 — Additional file 2. [file 12889_2022_13010_MOESM2_ESM.docx]

**Cross-Model Precision Prenatal HV Project Model Survey 2***

**Overview**

Thank you for completing the Cross-Model Precision Prenatal HV Project Model Survey 1.

The purpose of Survey 2 is to learn how your model views home visitors’ use of each of 23 techniques to promote those behaviors to reduce each of the risk factors.

Each model’s version of Survey 2 is tailored to its responses in Survey 1. Thus, your Survey 2 is limited to the XX behaviors that you indicated your model requires or recommends to reduce one or more risk factors and to the XX risk factors for low birthweight and preterm birth that you indicated are a priority for your model.

Your model’s version of Survey 2 has XX tables. There is one table for each behavior your model requires or recommends that home visitors aim to promote.

**The following represents the full set of all possible questions in Survey 2. Survey 2 was customized according to each model based on their responses to surveys 1 so that only priority risks (low, moderate, or high) and endorsed behaviors (required or recommended) were included.*

**A**. **Techniques to Promote Engaging in Physical Activity**

In Survey 1, you indicated that your model requires or recommends that home visitors promote expectant women to engage in physical activity as a way to reduce XX risk factors for low birthweight and preterm birth.

The table below has a column for each of these XX risk factors and a row for each of 23 techniques a home visitor might use to promote an expectant woman to engage in physical activity.

In each cell of the table, please indicate your model’s expectation of home visitors for using each technique to promote expectant women to engage in physical activity to reduce each of the risk factors.

Your model might have exactly the same expectation for using a technique for all of the risk factors, or it might have different expectations, depending on the risk factor. This table will allow us to learn whether models have different expectations for using a particular technique to promote expectant women to engage in physical activity, depending on the risk factor that is to be reduced.

| Please indicate your model’s expectation of home visitors for using each technique to promote expectant women **to engage in physical activity** to reduce each of the risk factors for low birthweight and preterm birth.  Note: Check ‘Required’ if you expect home visitors to use any strategy within the technique. You do not have to expect visitors to use all strategies within the technique to check ‘Required’. | | | | |
| --- | --- | --- | --- | --- |
|  | **Risk Factors** | | | |
| **Technique** | **High Blood Pressure** | **Diabetes** | **High Stress** | **Depression** |
| Assess readiness for change^[[1]](#footnote-1)^ | Select response | Select response | Select response | Select response |
| Goals & planning^[[2]](#footnote-2)^ | Select response | Select response | Select response | Select response |
| Monitoring & feedback^[[3]](#footnote-3)^ | Select response | Select response | Select response | Select response |
| Provide social support^[[4]](#footnote-4)^ | Select response | Select response | Select response | Select response |
| Suggest or arrange social support^[[5]](#footnote-5)^ | Select response | Select response | Select response | Select response |
| Natural consequences^[[6]](#footnote-6)^ | Select response | Select response | Select response | Select response |
| Shape knowledge of behavior^[[7]](#footnote-7)^ | Select response | Select response | Select response | Select response |
| Antecedents^[[8]](#footnote-8)^ | Select response | Select response | Select response | Select response |
| Behavior observation^[[9]](#footnote-9)^ | Select response | Select response | Select response | Select response |
| Associations to promote wanted behavior^[[10]](#footnote-10)^ | Select response | Select response | Select response | Select response |
| Associations to deter unwanted behavior^[[11]](#footnote-11)^ | Select response | Select response | Select response | Select response |
| Repetition & substitution^[[12]](#footnote-12)^ | Select response | Select response | Select response | Select response |
| Comparison of outcomes^[[13]](#footnote-13)^ | Select response | Select response | Select response | Select response |
| Credible source^[[14]](#footnote-14)^ | Select response | Select response | Select response | Select response |
| Incentives & rewards^[[15]](#footnote-15)^ | Select response | Select response | Select response | Select response |
| Scheduled consequences^[[16]](#footnote-16)^ | Select response | Select response | Select response | Select response |
| Mental regulation^[[17]](#footnote-17)^ | Select response | Select response | Select response | Select response |
| Identity as example to others^[[18]](#footnote-18)^ | Select response | Select response | Select response | Select response |
| Self-identity^[[19]](#footnote-19)^ | Select response | Select response | Select response | Select response |
| Self-belief^[[20]](#footnote-20)^ | Select response | Select response | Select response | Select response |
| Referral & linkage^[[21]](#footnote-21)^ | Select response | Select response | Select response | Select response |
| Monitoring & follow-up of referral^[[22]](#footnote-22)^ | Select response | Select response | Select response | Select response |
| Coordination with other services^[[23]](#footnote-23)^ | Select response | Select response | Select response | Select response |

**B. Techniques to Promote Adherence to a Healthy Diet**

In Survey 1, you indicated that your model requires or recommends that home visitors promote expectant women’s adherence to a healthy diet as a way to reduce XX risk factors for low birthweight and preterm birth.

The table below has a column for each of these XX risk factors and a row for each of 23 techniques a home visitor might use to promote an expectant woman’s adherence to a healthy diet.

In each cell of the table, please indicate your model’s expectation of home visitors for using each technique to promote expectant women’s adherence to a healthy diet to reduce each of the risk factors.

Your model might have exactly the same expectation for using a technique for all of the risk factors, or it might have different expectations, depending on the risk factor. This table will allow us to learn whether models have different expectations for using a particular technique to promote expectant women’s adherence to a healthy diet, depending on the risk factor that is to be reduced.

| Please indicate your model’s expectation of home visitors for using each technique to promote expectant women’s **adherence to a healthy diet** to reduce each of the risk factors for low birthweight and preterm birth.  Note: Check ‘Required’ if you expect home visitors to use any strategy within the technique. You do not have to expect visitors to use all strategies within the technique to check ‘Required’. | | |
| --- | --- | --- |
|  | **Risk Factors** | |
| **Technique** | **High Blood Pressure** | **Diabetes** |
| Assess readiness for change^[[24]](#footnote-24)^ | Select response | Select response |
| Goals & planning^[[25]](#footnote-25)^ | Select response | Select response |
| Monitoring & feedback^[[26]](#footnote-26)^ | Select response | Select response |
| Provide social support^[[27]](#footnote-27)^ | Select response | Select response |
| Suggest or arrange social support^[[28]](#footnote-28)^ | Select response | Select response |
| Natural consequences^[[29]](#footnote-29)^ | Select response | Select response |
| Shape knowledge of behavior^[[30]](#footnote-30)^ | Select response | Select response |
| Antecedents^[[31]](#footnote-31)^ | Select response | Select response |
| Behavior observation^[[32]](#footnote-32)^ | Select response | Select response |
| Associations to promote wanted behavior^[[33]](#footnote-33)^ | Select response | Select response |
| Associations to deter unwanted behavior^[[34]](#footnote-34)^ | Select response | Select response |
| Repetition & substitution^[[35]](#footnote-35)^ | Select response | Select response |
| Comparison of outcomes^[[36]](#footnote-36)^ | Select response | Select response |
| Credible source^[[37]](#footnote-37)^ | Select response | Select response |
| Incentives & rewards^[[38]](#footnote-38)^ | Select response | Select response |
| Scheduled consequences^[[39]](#footnote-39)^ | Select response | Select response |
| Mental regulation^[[40]](#footnote-40)^ | Select response | Select response |
| Identity as example to others^[[41]](#footnote-41)^ | Select response | Select response |
| Self-identity^[[42]](#footnote-42)^ | Select response | Select response |
| Self-belief^[[43]](#footnote-43)^ | Select response | Select response |
| Referral & linkage^[[44]](#footnote-44)^ | Select response | Select response |
| Monitoring & follow-up of referral^[[45]](#footnote-45)^ | Select response | Select response |
| Coordination with other services^[[46]](#footnote-46)^ | Select response | Select response |

**C. Techniques to Promote Stopping or Reducing Tobacco Use**

In Survey 1, you indicated that your model requires or recommends that home visitors promote expectant women to stop or reduce tobacco use as a way to reduce XX risk factors for low birthweight and preterm birth.

The table below has a column for each of these XX risk factors and a row for each of XX techniques a home visitor might use to promote an expectant woman to stop or reduce tobacco use.

In each cell of the table, please indicate your model’s expectation of home visitors for using each technique to promote expectant women to stop or reduce tobacco use to reduce each of the risk factors.

Your model might have exactly the same expectation for using a technique for all of the risk factors, or it might have different expectations, depending on the risk factor. This table will allow us to learn whether models have different expectations for using a particular technique to promote expectant women to stop or reduce tobacco use, depending on the risk factor that is to be reduced.

| Please indicate your model’s expectation of home visitors for using each technique to promote expectant women **to stop or reduce tobacco use** to reduce each of the risk factors for low birthweight and preterm birth.  Note: Check ‘Required’ if you expect home visitors to use any strategy within the technique. You do not have to expect visitors to use all strategies within the technique to check ‘Required’. | | |
| --- | --- | --- |
|  | **Risk Factors** | |
| **Technique** | **High Blood Pressure** | **Tobacco Use** |
| Assess readiness for change^[[47]](#footnote-47)^ | Select response | Select response |
| Goals & planning^[[48]](#footnote-48)^ | Select response | Select response |
| Monitoring & feedback^[[49]](#footnote-49)^ | Select response | Select response |
| Provide social support^[[50]](#footnote-50)^ | Select response | Select response |
| Suggest or arrange social support^[[51]](#footnote-51)^ | Select response | Select response |
| Natural consequences^[[52]](#footnote-52)^ | Select response | Select response |
| Shape knowledge of behavior^[[53]](#footnote-53)^ | Select response | Select response |
| Antecedents^[[54]](#footnote-54)^ | Select response | Select response |
| Behavior observation^[[55]](#footnote-55)^ | Select response | Select response |
| Associations to promote wanted behavior^[[56]](#footnote-56)^ | Select response | Select response |
| Associations to deter unwanted behavior^[[57]](#footnote-57)^ | Select response | Select response |
| Repetition & substitution^[[58]](#footnote-58)^ | Select response | Select response |
| Comparison of outcomes^[[59]](#footnote-59)^ | Select response | Select response |
| Credible source^[[60]](#footnote-60)^ | Select response | Select response |
| Incentives & rewards^[[61]](#footnote-61)^ | Select response | Select response |
| Scheduled consequences^[[62]](#footnote-62)^ | Select response | Select response |
| Mental regulation^[[63]](#footnote-63)^ | Select response | Select response |
| Identity as example to others^[[64]](#footnote-64)^ | Select response | Select response |
| Self-identity^[[65]](#footnote-65)^ | Select response | Select response |
| Self-belief^[[66]](#footnote-66)^ | Select response | Select response |
| Referral & linkage^[[67]](#footnote-67)^ | Select response | Select response |
| Monitoring & follow-up of referral^[[68]](#footnote-68)^ | Select response | Select response |
| Coordination with other services^[[69]](#footnote-69)^ | Select response | Select response |

**D. Techniques to Promote Stopping or Reducing Alcohol Use**

In Survey 1, you indicated that your model requires or recommends that home visitors promote expectant women to stop or reduce alcohol use as a way to reduce XX risk factors for low birthweight and preterm birth.

The table below has a column for each of these XX risk factors and a row for each of 23 techniques a home visitor might use to promote an expectant woman to stop or reduce alcohol use.

In each cell of the table, please indicate your model’s expectation of home visitors for using each technique to promote expectant women to stop or reduce alcohol use to reduce each of the risk factors.

Your model might have exactly the same expectation for using a technique for all of the risk factors, or it might have different expectations, depending on the risk factor. This table will allow us to learn whether models have different expectations for using a particular technique to promote expectant women to stop or reduce alcohol use, depending on the risk factor that is to be reduced.

| Please indicate your model’s expectation of home visitors for using each technique to promote expectant women **to stop or reduce alcohol use** to reduce each of the risk factors for low birthweight and preterm birth.  Note: Check ‘Required’ if you expect home visitors to use any strategy within the technique. You do not have to expect visitors to use all strategies within the technique to check ‘Required’. | | |
| --- | --- | --- |
|  | **Risk Factors** | |
| **Technique** | **High Blood Pressure** | **Alcohol Use** |
| Assess readiness for change^[[70]](#footnote-70)^ | Select response | Select response |
| Goals & planning^[[71]](#footnote-71)^ | Select response | Select response |
| Monitoring & feedback^[[72]](#footnote-72)^ | Select response | Select response |
| Provide social support^[[73]](#footnote-73)^ | Select response | Select response |
| Suggest or arrange social support^[[74]](#footnote-74)^ | Select response | Select response |
| Natural consequences^[[75]](#footnote-75)^ | Select response | Select response |
| Shape knowledge of behavior^[[76]](#footnote-76)^ | Select response | Select response |
| Antecedents^[[77]](#footnote-77)^ | Select response | Select response |
| Behavior observation^[[78]](#footnote-78)^ | Select response | Select response |
| Associations to promote wanted behavior^[[79]](#footnote-79)^ | Select response | Select response |
| Associations to deter unwanted behavior^[[80]](#footnote-80)^ | Select response | Select response |
| Repetition & substitution^[[81]](#footnote-81)^ | Select response | Select response |
| Comparison of outcomes^[[82]](#footnote-82)^ | Select response | Select response |
| Credible source^[[83]](#footnote-83)^ | Select response | Select response |
| Incentives & rewards^[[84]](#footnote-84)^ | Select response | Select response |
| Scheduled consequences^[[85]](#footnote-85)^ | Select response | Select response |
| Mental regulation^[[86]](#footnote-86)^ | Select response | Select response |
| Identity as example to others^[[87]](#footnote-87)^ | Select response | Select response |
| Self-identity^[[88]](#footnote-88)^ | Select response | Select response |
| Self-belief^[[89]](#footnote-89)^ | Select response | Select response |
| Referral & linkage^[[90]](#footnote-90)^ | Select response | Select response |
| Monitoring & follow-up of referral^[[91]](#footnote-91)^ | Select response | Select response |
| Coordination with other services^[[92]](#footnote-92)^ | Select response | Select response |

**E. Techniques to Promote Stopping or Reducing Illicit Drug Use**

In Survey 1, you indicated that your model requires or recommends that home visitors promote expectant women to stop or reduce illicit drug use as a way to reduce XX risk factors for low birthweight and preterm birth.

The table below has a column for each of these XX risk factors and a row for each of 23 techniques a home visitor might use to promote an expectant woman to stop or reduce illicit drug use.

In each cell of the table, please indicate your model’s expectation of home visitors for using each technique to promote expectant women to stop or reduce illicit drug use to reduce each of the risk factors.

Your model might have exactly the same expectation for using a technique for all of the risk factors, or it might have different expectations, depending on the risk factor. This table will allow us to learn whether models have different expectations for using a particular technique to promote expectant women to stop or reduce illicit drug use, depending on the risk factor that is to be reduced.

| Please indicate your model’s expectation of home visitors for using each technique to promote expectant women **to stop or reduce illicit drug use** to reduce each of the risk factors for low birthweight and preterm birth.  Note: Check ‘Required’ if you expect home visitors to use any strategy within the technique. You do not have to expect visitors to use all strategies within the technique to check ‘Required’. | |
| --- | --- |
|  | **Risk Factor** |
| **Technique** | **Illicit Drug Use** |
| Assess readiness for change^[[93]](#footnote-93)^ | Select response |
| Goals & planning^[[94]](#footnote-94)^ | Select response |
| Monitoring & feedback^[[95]](#footnote-95)^ | Select response |
| Provide social support^[[96]](#footnote-96)^ | Select response |
| Suggest or arrange social support^[[97]](#footnote-97)^ | Select response |
| Natural consequences^[[98]](#footnote-98)^ | Select response |
| Shape knowledge of behavior^[[99]](#footnote-99)^ | Select response |
| Antecedents^[[100]](#footnote-100)^ | Select response |
| Behavior observation^[[101]](#footnote-101)^ | Select response |
| Associations to promote wanted behavior^[[102]](#footnote-102)^ | Select response |
| Associations to deter unwanted behavior^[[103]](#footnote-103)^ | Select response |
| Repetition & substitution^[[104]](#footnote-104)^ | Select response |
| Comparison of outcomes^[[105]](#footnote-105)^ | Select response |
| Credible source^[[106]](#footnote-106)^ | Select response |
| Incentives & rewards^[[107]](#footnote-107)^ | Select response |
| Scheduled consequences^[[108]](#footnote-108)^ | Select response |
| Mental regulation^[[109]](#footnote-109)^ | Select response |
| Identity as example to others^[[110]](#footnote-110)^ | Select response |
| Self-identity^[[111]](#footnote-111)^ | Select response |
| Self-belief^[[112]](#footnote-112)^ | Select response |
| Referral & linkage^[[113]](#footnote-113)^ | Select response |
| Monitoring & follow-up of referral^[[114]](#footnote-114)^ | Select response |
| Coordination with other services^[[115]](#footnote-115)^ | Select response |

**F. Techniques to Promote Self-monitoring of Physiologic Indicators**

In Survey 1, you indicated that your model requires or recommends that home visitors promote expectant women to self-monitor physiologic indicators as a way to reduce XX risk factors for low birthweight and preterm birth.

The table below has a column for each of these XX risk factors and a row for each of 23 techniques a home visitor might use to promote an expectant woman to self-monitor physiologic indicators.

In each cell of the table, please indicate your model’s expectation of home visitors for using each technique to promote expectant women to self-monitor physiologic indicators to reduce each of the risk factors.

Your model might have exactly the same expectation for using a technique for all of the risk factors, or it might have different expectations, depending on the risk factor. This table will allow us to learn whether models have different expectations for using a particular technique to promote expectant women to self-monitor physiologic indicators, depending on the risk factor that is to be reduced.

| Please indicate your model’s expectation of home visitors for using each technique to promote expectant women **to self-monitor physiologic indicators** to reduce each of the risk factors for low birthweight and preterm birth.  Note: Check ‘Required’ if you expect home visitors to use any strategy within the technique. You do not have to expect visitors to use all strategies within the technique to check ‘Required’. | | |
| --- | --- | --- |
|  | **Risk Factors** | |
| **Technique** | **High Blood Pressure** | **Diabetes** |
| Assess readiness for change^[[116]](#footnote-116)^ | Select response | Select response |
| Goals & planning^[[117]](#footnote-117)^ | Select response | Select response |
| Monitoring & feedback^[[118]](#footnote-118)^ | Select response | Select response |
| Provide social support^[[119]](#footnote-119)^ | Select response | Select response |
| Suggest or arrange social support^[[120]](#footnote-120)^ | Select response | Select response |
| Natural consequences^[[121]](#footnote-121)^ | Select response | Select response |
| Shape knowledge of behavior^[[122]](#footnote-122)^ | Select response | Select response |
| Antecedents^[[123]](#footnote-123)^ | Select response | Select response |
| Behavior observation^[[124]](#footnote-124)^ | Select response | Select response |
| Associations to promote wanted behavior^[[125]](#footnote-125)^ | Select response | Select response |
| Associations to deter unwanted behavior^[[126]](#footnote-126)^ | Select response | Select response |
| Repetition & substitution^[[127]](#footnote-127)^ | Select response | Select response |
| Comparison of outcomes^[[128]](#footnote-128)^ | Select response | Select response |
| Credible source^[[129]](#footnote-129)^ | Select response | Select response |
| Incentives & rewards^[[130]](#footnote-130)^ | Select response | Select response |
| Scheduled consequences^[[131]](#footnote-131)^ | Select response | Select response |
| Mental regulation^[[132]](#footnote-132)^ | Select response | Select response |
| Identity as example to others^[[133]](#footnote-133)^ | Select response | Select response |
| Self-identity^[[134]](#footnote-134)^ | Select response | Select response |
| Self-belief^[[135]](#footnote-135)^ | Select response | Select response |
| Referral & linkage^[[136]](#footnote-136)^ | Select response | Select response |
| Monitoring & follow-up of referral^[[137]](#footnote-137)^ | Select response | Select response |
| Coordination with other services^[[138]](#footnote-138)^ | Select response | Select response |

**G. Techniques to Promote Adherence to Prenatal Care Provider Schedule**

In Survey 1, you indicated that your model requires or recommends that home visitors promote expectant women’s adherence to prenatal care provider visit schedule as a way to reduce XX risk factors for low birthweight and preterm birth.

The table below has a column for each of these XX risk factors and a row for each of 23 techniques a home visitor might use to promote an expectant woman’s adherence to prenatal care provider visit schedule.

In each cell of the table, please indicate your model’s expectation of home visitors for using each technique to promote expectant women’s adherence to prenatal care provider visit schedule to reduce each of the risk factors.

Your model might have exactly the same expectation for using a technique for all of the risk factors, or it might have different expectations, depending on the risk factor. This table will allow us to learn whether models have different expectations for using a particular technique to promote expectant women’s adherence to prenatal care provider visit schedule, depending on the risk factor that is to be reduced.

| Please indicate your model’s expectation of home visitors for using each technique to promote expectant women’s **adherence to prenatal care provider visit schedule** to reduce each of the risk factors for low birthweight and preterm birth.  Note: Check ‘Required’ if you expect home visitors to use any strategy within the technique. You do not have to expect visitors to use all strategies within the technique to check ‘Required’. | | | | |
| --- | --- | --- | --- | --- |
|  | **Risk Factors** | | | |
| **Technique** | **High Blood Pressure** | **Diabetes** | **Depression** | **Inadequate Prenatal Care** |
| Assess readiness for change^[[139]](#footnote-139)^ | Select response | Select response | Select response | Select response |
| Goals & planning^[[140]](#footnote-140)^ | Select response | Select response | Select response | Select response |
| Monitoring & feedback^[[141]](#footnote-141)^ | Select response | Select response | Select response | Select response |
| Provide social support^[[142]](#footnote-142)^ | Select response | Select response | Select response | Select response |
| Suggest or arrange social support^[[143]](#footnote-143)^ | Select response | Select response | Select response | Select response |
| Natural consequences^[[144]](#footnote-144)^ | Select response | Select response | Select response | Select response |
| Shape knowledge of behavior^[[145]](#footnote-145)^ | Select response | Select response | Select response | Select response |
| Antecedents^[[146]](#footnote-146)^ | Select response | Select response | Select response | Select response |
| Behavior observation^[[147]](#footnote-147)^ | Select response | Select response | Select response | Select response |
| Associations to promote wanted behavior^[[148]](#footnote-148)^ | Select response | Select response | Select response | Select response |
| Associations to deter unwanted behavior^[[149]](#footnote-149)^ | Select response | Select response | Select response | Select response |
| Repetition & substitution^[[150]](#footnote-150)^ | Select response | Select response | Select response | Select response |
| Comparison of outcomes^[[151]](#footnote-151)^ | Select response | Select response | Select response | Select response |
| Credible source^[[152]](#footnote-152)^ | Select response | Select response | Select response | Select response |
| Incentives & rewards^[[153]](#footnote-153)^ | Select response | Select response | Select response | Select response |
| Scheduled consequences^[[154]](#footnote-154)^ | Select response | Select response | Select response | Select response |
| Mental regulation^[[155]](#footnote-155)^ | Select response | Select response | Select response | Select response |
| Identity as example to others^[[156]](#footnote-156)^ | Select response | Select response | Select response | Select response |
| Self-identity^[[157]](#footnote-157)^ | Select response | Select response | Select response | Select response |
| Self-belief^[[158]](#footnote-158)^ | Select response | Select response | Select response | Select response |
| Referral & linkage^[[159]](#footnote-159)^ | Select response | Select response | Select response | Select response |
| Monitoring & follow-up of referral^[[160]](#footnote-160)^ | Select response | Select response | Select response | Select response |
| Coordination with other services^[[161]](#footnote-161)^ | Select response | Select response | Select response | Select response |

**H. Techniques to Promote Alerting Prenatal Care Provider to Warning Signs**

In Survey 1, you indicated that your model requires or recommends that home visitors promote expectant women to alert prenatal care provider to warning signs as a way to reduce XX risk factors for low birthweight and preterm birth.

The table below has a column for each of these XX risk factors and a row for each of 23 techniques a home visitor might use to promote an expectant woman to alert prenatal care provider to warning signs.

In each cell of the table, please indicate your model’s expectation of home visitors for using each technique to promote expectant women to alert prenatal care provider to warning signs to reduce each of the risk factors.

Your model might have exactly the same expectation for using a technique for all of the risk factors, or it might have different expectations, depending on the risk factor. This table will allow us to learn whether models have different expectations for using a particular technique to promote expectant women to alert prenatal care provider to warning signs, depending on the risk factor that is to be reduced.

| Please indicate your model’s expectation of home visitors for using each technique to promote expectant women **to alert prenatal care provider to warning signs** to reduce each of the risk factors for low birthweight and preterm birth.  Note: Check ‘Required’ if you expect home visitors to use any strategy within the technique. You do not have to expect visitors to use all strategies within the technique to check ‘Required’. | | | |
| --- | --- | --- | --- |
|  | **Risk Factors** | | |
| **Technique** | **High Blood Pressure** | **Diabetes** | **Depression** |
| Assess readiness for change^[[162]](#footnote-162)^ | Select response | Select response | Select response |
| Goals & planning^[[163]](#footnote-163)^ | Select response | Select response | Select response |
| Monitoring & feedback^[[164]](#footnote-164)^ | Select response | Select response | Select response |
| Provide social support^[[165]](#footnote-165)^ | Select response | Select response | Select response |
| Suggest or arrange social support^[[166]](#footnote-166)^ | Select response | Select response | Select response |
| Natural consequences^[[167]](#footnote-167)^ | Select response | Select response | Select response |
| Shape knowledge of behavior^[[168]](#footnote-168)^ | Select response | Select response | Select response |
| Antecedents^[[169]](#footnote-169)^ | Select response | Select response | Select response |
| Behavior observation^[[170]](#footnote-170)^ | Select response | Select response | Select response |
| Associations to promote wanted behavior^[[171]](#footnote-171)^ | Select response | Select response | Select response |
| Associations to deter unwanted behavior^[[172]](#footnote-172)^ | Select response | Select response | Select response |
| Repetition & substitution^[[173]](#footnote-173)^ | Select response | Select response | Select response |
| Comparison of outcomes^[[174]](#footnote-174)^ | Select response | Select response | Select response |
| Credible source^[[175]](#footnote-175)^ | Select response | Select response | Select response |
| Incentives & rewards^[[176]](#footnote-176)^ | Select response | Select response | Select response |
| Scheduled consequences^[[177]](#footnote-177)^ | Select response | Select response | Select response |
| Mental regulation^[[178]](#footnote-178)^ | Select response | Select response | Select response |
| Identity as example to others^[[179]](#footnote-179)^ | Select response | Select response | Select response |
| Self-identity^[[180]](#footnote-180)^ | Select response | Select response | Select response |
| Self-belief^[[181]](#footnote-181)^ | Select response | Select response | Select response |
| Referral & linkage^[[182]](#footnote-182)^ | Select response | Select response | Select response |
| Monitoring & follow-up of referral^[[183]](#footnote-183)^ | Select response | Select response | Select response |
| Coordination with other services^[[184]](#footnote-184)^ | Select response | Select response | Select response |

**I. Techniques to Promote Condom Use**

In Survey 1, you indicated that your model requires or recommends that home visitors promote expectant women to use condoms as a way to reduce XX risk factors for low birthweight and preterm birth.

The table below has a column for each of these XX risk factors and a row for each of 23 techniques a home visitor might use to promote an expectant woman to use condoms.

In each cell of the table, please indicate your model’s expectation of home visitors for using each technique to promote expectant women to use condoms to reduce each of the risk factors.

Your model might have exactly the same expectation for using a technique for all of the risk factors, or it might have different expectations, depending on the risk factor. This table will allow us to learn whether models have different expectations for using a particular technique to promote expectant women to use condoms, depending on the risk factor that is to be reduced.

| Please indicate your model’s expectation of home visitors for using each technique to promote expectant women **to use condoms** to reduce each of the risk factors for low birthweight and preterm birth.  Note: Check ‘Required’ if you expect home visitors to use any strategy within the technique. You do not have to expect visitors to use all strategies within the technique to check ‘Required’. | |
| --- | --- |
|  | **Risk Factor** |
| **Technique** | **Infection** |
| Assess readiness for change^[[185]](#footnote-185)^ | Select response |
| Goals & planning^[[186]](#footnote-186)^ | Select response |
| Monitoring & feedback^[[187]](#footnote-187)^ | Select response |
| Provide social support^[[188]](#footnote-188)^ | Select response |
| Suggest or arrange social support^[[189]](#footnote-189)^ | Select response |
| Natural consequences^[[190]](#footnote-190)^ | Select response |
| Shape knowledge of behavior^[[191]](#footnote-191)^ | Select response |
| Antecedents^[[192]](#footnote-192)^ | Select response |
| Behavior observation^[[193]](#footnote-193)^ | Select response |
| Associations to promote wanted behavior^[[194]](#footnote-194)^ | Select response |
| Associations to deter unwanted behavior^[[195]](#footnote-195)^ | Select response |
| Repetition & substitution^[[196]](#footnote-196)^ | Select response |
| Comparison of outcomes^[[197]](#footnote-197)^ | Select response |
| Credible source^[[198]](#footnote-198)^ | Select response |
| Incentives & rewards^[[199]](#footnote-199)^ | Select response |
| Scheduled consequences^[[200]](#footnote-200)^ | Select response |
| Mental regulation^[[201]](#footnote-201)^ | Select response |
| Identity as example to others^[[202]](#footnote-202)^ | Select response |
| Self-identity^[[203]](#footnote-203)^ | Select response |
| Self-belief^[[204]](#footnote-204)^ | Select response |
| Referral & linkage^[[205]](#footnote-205)^ | Select response |
| Monitoring & follow-up of referral^[[206]](#footnote-206)^ | Select response |
| Coordination with other services^[[207]](#footnote-207)^ | Select response |

**J. Techniques to Promote Adherence to Prescribed Medication Regimens**

In Survey 1, you indicated that your model requires or recommends that home visitors promote expectant women’s adherence to prescribed medication regimen as a way to reduce XX risk factors for low birthweight and preterm birth.

The table below has a column for each of these XX risk factors and a row for each of 23 techniques a home visitor might use to promote an expectant woman’s adherence to prescribed medication regimen.

In each cell of the table, please indicate your model’s expectation of home visitors for using each technique to promote expectant women’s adherence to prescribed medication regimen to reduce each of the risk factors.

Your model might have exactly the same expectation for using a technique for all of the risk factors, or it might have different expectations, depending on the risk factor. This table will allow us to learn whether models have different expectations for using a particular technique to promote expectant women’s adherence to prescribed medication regimen, depending on the risk factor that is to be reduced.

| Please indicate your model’s expectation of home visitors for using each technique to promote expectant women’s **adherence to prescribed medication regimen** to reduce each of the risk factors for low birthweight and preterm birth.  Note: Check ‘Required’ if you expect home visitors to use any strategy within the technique. You do not have to expect visitors to use all strategies within the technique to check ‘Required’. | | | | | | |
| --- | --- | --- | --- | --- | --- | --- |
|  | **Risk Factors** | | | | | |
| **Technique** | **High Blood Pressure** | **Diabetes** | **Infection** | **Depression** | **Tobacco Use** | **Illicit Drug Use** |
| Assess readiness for change^[[208]](#footnote-208)^ | Select response | Select response | Select response | Select response | Select response | Select response |
| Goals & planning^[[209]](#footnote-209)^ | Select response | Select response | Select response | Select response | Select response | Select response |
| Monitoring & feedback^[[210]](#footnote-210)^ | Select response | Select response | Select response | Select response | Select response | Select response |
| Provide social support^[[211]](#footnote-211)^ | Select response | Select response | Select response | Select response | Select response | Select response |
| Suggest or arrange social support^[[212]](#footnote-212)^ | Select response | Select response | Select response | Select response | Select response | Select response |
| Natural consequences^[[213]](#footnote-213)^ | Select response | Select response | Select response | Select response | Select response | Select response |
| Shape knowledge of behavior^[[214]](#footnote-214)^ | Select response | Select response | Select response | Select response | Select response | Select response |
| Antecedents^[[215]](#footnote-215)^ | Select response | Select response | Select response | Select response | Select response | Select response |
| Behavior observation^[[216]](#footnote-216)^ | Select response | Select response | Select response | Select response | Select response | Select response |
| Associations to promote wanted behavior^[[217]](#footnote-217)^ | Select response | Select response | Select response | Select response | Select response | Select response |
| Associations to deter unwanted behavior^[[218]](#footnote-218)^ | Select response | Select response | Select response | Select response | Select response | Select response |
| Repetition & substitution^[[219]](#footnote-219)^ | Select response | Select response | Select response | Select response | Select response | Select response |
| Comparison of outcomes^[[220]](#footnote-220)^ | Select response | Select response | Select response | Select response | Select response | Select response |
| Credible source^[[221]](#footnote-221)^ | Select response | Select response | Select response | Select response | Select response | Select response |
| Incentives & rewards^[[222]](#footnote-222)^ | Select response | Select response | Select response | Select response | Select response | Select response |
| Scheduled consequences^[[223]](#footnote-223)^ | Select response | Select response | Select response | Select response | Select response | Select response |
| Mental regulation^[[224]](#footnote-224)^ | Select response | Select response | Select response | Select response | Select response | Select response |
| Identity as example to others^[[225]](#footnote-225)^ | Select response | Select response | Select response | Select response | Select response | Select response |
| Self-identity^[[226]](#footnote-226)^ | Select response | Select response | Select response | Select response | Select response | Select response |
| Self-belief^[[227]](#footnote-227)^ | Select response | Select response | Select response | Select response | Select response | Select response |
| Referral & linkage^[[228]](#footnote-228)^ | Select response | Select response | Select response | Select response | Select response | Select response |
| Monitoring & follow-up of referral^[[229]](#footnote-229)^ | Select response | Select response | Select response | Select response | Select response | Select response |
| Coordination with other services^[[230]](#footnote-230)^ | Select response | Select response | Select response | Select response | Select response | Select response |

**K. Techniques to Promote Engaging in Stress Reduction Activities**

In Survey 1, you indicated that your model requires or recommends that home visitors promote expectant women to engage in stress reduction activities as a way to reduce XX risk factors for low birthweight and preterm birth.

The table below has a column for each of these XX risk factors and a row for each of 23 techniques a home visitor might use to promote an expectant woman to engage in stress reduction activities.

In each cell of the table, please indicate your model’s expectation of home visitors for using each technique to promote expectant women to engage in stress reduction activities to reduce each of the risk factors.

Your model might have exactly the same expectation for using a technique for all of the risk factors, or it might have different expectations, depending on the risk factor. This table will allow us to learn whether models have different expectations for using a particular technique to promote expectant women to engage in stress reduction activities, depending on the risk factor that is to be reduced.

| Please indicate your model’s expectation of home visitors for using each technique to promote expectant women **to engage in stress reduction activities** to reduce each of the risk factors for low birthweight and preterm birth.  Note: Check ‘Required’ if you expect home visitors to use any strategy within the technique. You do not have to expect visitors to use all strategies within the technique to check ‘Required’. | | | | | | |
| --- | --- | --- | --- | --- | --- | --- |
|  | **Risk Factors** | | | | | |
| **Technique** | **IPV** | **High Stress** | **Depression** | **Tobacco Use** | **Alcohol Use** | **Illicit Drug Use** |
| Assess readiness for change^[[231]](#footnote-231)^ | Select response | Select response | Select response | Select response | Select response | Select response |
| Goals & planning^[[232]](#footnote-232)^ | Select response | Select response | Select response | Select response | Select response | Select response |
| Monitoring & feedback^[[233]](#footnote-233)^ | Select response | Select response | Select response | Select response | Select response | Select response |
| Provide social support^[[234]](#footnote-234)^ | Select response | Select response | Select response | Select response | Select response | Select response |
| Suggest or arrange social support^[[235]](#footnote-235)^ | Select response | Select response | Select response | Select response | Select response | Select response |
| Natural consequences^[[236]](#footnote-236)^ | Select response | Select response | Select response | Select response | Select response | Select response |
| Shape knowledge of behavior^[[237]](#footnote-237)^ | Select response | Select response | Select response | Select response | Select response | Select response |
| Antecedents^[[238]](#footnote-238)^ | Select response | Select response | Select response | Select response | Select response | Select response |
| Behavior observation^[[239]](#footnote-239)^ | Select response | Select response | Select response | Select response | Select response | Select response |
| Associations to promote wanted behavior^[[240]](#footnote-240)^ | Select response | Select response | Select response | Select response | Select response | Select response |
| Associations to deter unwanted behavior^[[241]](#footnote-241)^ | Select response | Select response | Select response | Select response | Select response | Select response |
| Repetition & substitution^[[242]](#footnote-242)^ | Select response | Select response | Select response | Select response | Select response | Select response |
| Comparison of outcomes^[[243]](#footnote-243)^ | Select response | Select response | Select response | Select response | Select response | Select response |
| Credible source^[[244]](#footnote-244)^ | Select response | Select response | Select response | Select response | Select response | Select response |
| Incentives & rewards^[[245]](#footnote-245)^ | Select response | Select response | Select response | Select response | Select response | Select response |
| Scheduled consequences^[[246]](#footnote-246)^ | Select response | Select response | Select response | Select response | Select response | Select response |
| Mental regulation^[[247]](#footnote-247)^ | Select response | Select response | Select response | Select response | Select response | Select response |
| Identity as example to others^[[248]](#footnote-248)^ | Select response | Select response | Select response | Select response | Select response | Select response |
| Self-identity^[[249]](#footnote-249)^ | Select response | Select response | Select response | Select response | Select response | Select response |
| Self-belief^[[250]](#footnote-250)^ | Select response | Select response | Select response | Select response | Select response | Select response |
| Referral & linkage^[[251]](#footnote-251)^ | Select response | Select response | Select response | Select response | Select response | Select response |
| Monitoring & follow-up of referral^[[252]](#footnote-252)^ | Select response | Select response | Select response | Select response | Select response | Select response |
| Coordination with other services^[[253]](#footnote-253)^ | Select response | Select response | Select response | Select response | Select response | Select response |

**L. Techniques to Promote Social Support Use**

In Survey 1, you indicated that your model requires or recommends that home visitors promote expectant women to use social supports as a way to reduce XX risk factors for low birthweight and preterm birth.

The table below has a column for each of these XX risk factors and a row for each of 23 techniques a home visitor might use to promote an expectant woman to use social supports.

In each cell of the table, please indicate your model’s expectation of home visitors for using each technique to promote expectant women to use social supports to reduce each of the risk factors.

Your model might have exactly the same expectation for using a technique for all of the risk factors, or it might have different expectations, depending on the risk factor. This table will allow us to learn whether models have different expectations for using a particular technique to promote expectant women to use social supports, depending on the risk factor that is to be reduced.

| Please indicate your model’s expectation of home visitors for using each technique to promote expectant women **to use social supports** to reduce each of the risk factors for low birthweight and preterm birth.  Note: Check ‘Required’ if you expect home visitors to use any strategy within the technique. You do not have to expect visitors to use all strategies within the technique to check ‘Required’. | | | | | | |
| --- | --- | --- | --- | --- | --- | --- |
|  | **Risk Factors** | | | | | |
| **Technique** | **IPV** | **High Stress** | **Depression** | **Tobacco Use** | **Alcohol Use** | **Illicit Drug Use** |
| Assess readiness for change^[[254]](#footnote-254)^ | Select response | Select response | Select response | Select response | Select response | Select response |
| Goals & planning^[[255]](#footnote-255)^ | Select response | Select response | Select response | Select response | Select response | Select response |
| Monitoring & feedback^[[256]](#footnote-256)^ | Select response | Select response | Select response | Select response | Select response | Select response |
| Provide social support^[[257]](#footnote-257)^ | Select response | Select response | Select response | Select response | Select response | Select response |
| Suggest or arrange social support^[[258]](#footnote-258)^ | Select response | Select response | Select response | Select response | Select response | Select response |
| Natural consequences^[[259]](#footnote-259)^ | Select response | Select response | Select response | Select response | Select response | Select response |
| Shape knowledge of behavior^[[260]](#footnote-260)^ | Select response | Select response | Select response | Select response | Select response | Select response |
| Antecedents^[[261]](#footnote-261)^ | Select response | Select response | Select response | Select response | Select response | Select response |
| Behavior observation^[[262]](#footnote-262)^ | Select response | Select response | Select response | Select response | Select response | Select response |
| Associations to promote wanted behavior^[[263]](#footnote-263)^ | Select response | Select response | Select response | Select response | Select response | Select response |
| Associations to deter unwanted behavior^[[264]](#footnote-264)^ | Select response | Select response | Select response | Select response | Select response | Select response |
| Repetition & substitution^[[265]](#footnote-265)^ | Select response | Select response | Select response | Select response | Select response | Select response |
| Comparison of outcomes^[[266]](#footnote-266)^ | Select response | Select response | Select response | Select response | Select response | Select response |
| Credible source^[[267]](#footnote-267)^ | Select response | Select response | Select response | Select response | Select response | Select response |
| Incentives & rewards^[[268]](#footnote-268)^ | Select response | Select response | Select response | Select response | Select response | Select response |
| Scheduled consequences^[[269]](#footnote-269)^ | Select response | Select response | Select response | Select response | Select response | Select response |
| Mental regulation^[[270]](#footnote-270)^ | Select response | Select response | Select response | Select response | Select response | Select response |
| Identity as example to others^[[271]](#footnote-271)^ | Select response | Select response | Select response | Select response | Select response | Select response |
| Self-identity^[[272]](#footnote-272)^ | Select response | Select response | Select response | Select response | Select response | Select response |
| Self-belief^[[273]](#footnote-273)^ | Select response | Select response | Select response | Select response | Select response | Select response |
| Referral & linkage^[[274]](#footnote-274)^ | Select response | Select response | Select response | Select response | Select response | Select response |
| Monitoring & follow-up of referral^[[275]](#footnote-275)^ | Select response | Select response | Select response | Select response | Select response | Select response |
| Coordination with other services^[[276]](#footnote-276)^ | Select response | Select response | Select response | Select response | Select response | Select response |

**M. Techniques to Promote Developing a Safety Plan**

In Survey 1, you indicated that your model requires or recommends that home visitors promote expectant women to develop a safety plan as a way to reduce XX risk factors for low birthweight and preterm birth.

The table below has a column for each of these XX risk factors and a row for each of 23 techniques a home visitor might use to promote an expectant woman to develop a safety plan.

In each cell of the table, please indicate your model’s expectation of home visitors for using each technique to promote expectant women to develop a safety plan to reduce each of the risk factors.

Your model might have exactly the same expectation for using a technique for all of the risk factors, or it might have different expectations, depending on the risk factor. This table will allow us to learn whether models have different expectations for using a particular technique to promote expectant women to develop a safety plan, depending on the risk factor that is to be reduced.

| Please indicate your model’s expectation of home visitors for using each technique to promote expectant women **to develop a safety plan** to reduce each of the risk factors for low birthweight and preterm birth.  Note: Check ‘Required’ if you expect home visitors to use any strategy within the technique. You do not have to expect visitors to use all strategies within the technique to check ‘Required’. | |
| --- | --- |
|  | **Risk Factor** |
| **Technique** | **IPV** |
| Assess readiness for change^[[277]](#footnote-277)^ | Select response |
| Goals & planning^[[278]](#footnote-278)^ | Select response |
| Monitoring & feedback^[[279]](#footnote-279)^ | Select response |
| Provide social support^[[280]](#footnote-280)^ | Select response |
| Suggest or arrange social support^[[281]](#footnote-281)^ | Select response |
| Natural consequences^[[282]](#footnote-282)^ | Select response |
| Shape knowledge of behavior^[[283]](#footnote-283)^ | Select response |
| Antecedents^[[284]](#footnote-284)^ | Select response |
| Behavior observation^[[285]](#footnote-285)^ | Select response |
| Associations to promote wanted behavior^[[286]](#footnote-286)^ | Select response |
| Associations to deter unwanted behavior^[[287]](#footnote-287)^ | Select response |
| Repetition & substitution^[[288]](#footnote-288)^ | Select response |
| Comparison of outcomes^[[289]](#footnote-289)^ | Select response |
| Credible source^[[290]](#footnote-290)^ | Select response |
| Incentives & rewards^[[291]](#footnote-291)^ | Select response |
| Scheduled consequences^[[292]](#footnote-292)^ | Select response |
| Mental regulation^[[293]](#footnote-293)^ | Select response |
| Identity as example to others^[[294]](#footnote-294)^ | Select response |
| Self-identity^[[295]](#footnote-295)^ | Select response |
| Self-belief^[[296]](#footnote-296)^ | Select response |
| Referral & linkage^[[297]](#footnote-297)^ | Select response |
| Monitoring & follow-up of referral^[[298]](#footnote-298)^ | Select response |
| Coordination with other services^[[299]](#footnote-299)^ | Select response |

**N. Techniques to Promote Engaging in Substance Use Treatment**

In Survey 1, you indicated that your model requires or recommends that home visitors promote expectant women to engage in substance use treatment as a way to reduce XX risk factors for low birthweight and preterm birth.

The table below has a column for each of these XX risk factors and a row for each of 23 techniques a home visitor might use to promote an expectant woman to engage in substance use treatment.

In each cell of the table, please indicate your model’s expectation of home visitors for using each technique to promote expectant women to engage in substance use treatment to reduce each of the risk factors.

Your model might have exactly the same expectation for using a technique for all of the risk factors, or it might have different expectations, depending on the risk factor. This table will allow us to learn whether models have different expectations for using a particular technique to promote expectant women to engage in substance use treatment, depending on the risk factor that is to be reduced.

| Please indicate your model’s expectation of home visitors for using each technique to promote expectant women **to engage in substance use treatment** to reduce each of the risk factors for low birthweight and preterm birth.  Note: Check ‘Required’ if you expect home visitors to use any strategy within the technique. You do not have to expect visitors to use all strategies within the technique to check ‘Required’. | |
| --- | --- |
|  | **Risk Factor** |
| **Technique** | **Illicit Drug Use** |
| Assess readiness for change^[[300]](#footnote-300)^ | Select response |
| Goals & planning^[[301]](#footnote-301)^ | Select response |
| Monitoring & feedback^[[302]](#footnote-302)^ | Select response |
| Provide social support^[[303]](#footnote-303)^ | Select response |
| Suggest or arrange social support^[[304]](#footnote-304)^ | Select response |
| Natural consequences^[[305]](#footnote-305)^ | Select response |
| Shape knowledge of behavior^[[306]](#footnote-306)^ | Select response |
| Antecedents^[[307]](#footnote-307)^ | Select response |
| Behavior observation^[[308]](#footnote-308)^ | Select response |
| Associations to promote wanted behavior^[[309]](#footnote-309)^ | Select response |
| Associations to deter unwanted behavior^[[310]](#footnote-310)^ | Select response |
| Repetition & substitution^[[311]](#footnote-311)^ | Select response |
| Comparison of outcomes^[[312]](#footnote-312)^ | Select response |
| Credible source^[[313]](#footnote-313)^ | Select response |
| Incentives & rewards^[[314]](#footnote-314)^ | Select response |
| Scheduled consequences^[[315]](#footnote-315)^ | Select response |
| Mental regulation^[[316]](#footnote-316)^ | Select response |
| Identity as example to others^[[317]](#footnote-317)^ | Select response |
| Self-identity^[[318]](#footnote-318)^ | Select response |
| Self-belief^[[319]](#footnote-319)^ | Select response |
| Referral & linkage^[[320]](#footnote-320)^ | Select response |
| Monitoring & follow-up of referral^[[321]](#footnote-321)^ | Select response |
| Coordination with other services^[[322]](#footnote-322)^ | Select response |

1. ^1^ Gather information about the expectant woman’s readiness to change the behavior. [↑](#footnote-ref-1)
2. Assist the expectant woman to: set a behavior change goal; develop a plan to meet the goal using strategies to overcome barriers and increase facilitators; review her progress toward the goal; modify the goal or plan as needed. [↑](#footnote-ref-2)
3. Monitor the expectant woman’s progress in changing the behavior; give feedback on that progress; establish ways for the expectant woman to self-monitor her progress. [↑](#footnote-ref-3)
4. Directly provide the expectant woman encouragement, emotional support or practical help to perform the behavior. [↑](#footnote-ref-4)
5. Suggest or assist the expectant woman to seek encouragement, emotional support or practical help to perform the behavior from a friend, relative, colleague, or group. [↑](#footnote-ref-5)
6. Provide written, verbal or visual information about the behavior’s health, emotional, social or environmental consequences; encourage her to assess her feelings after attempts to perform the behavior; raise her awareness of future regret about performing the unwanted behavior. [↑](#footnote-ref-6)
7. Provide information or instruction to shape the expectant woman’s knowledge of *how to perform the behavior*. This includes identification of behavioral ‘triggers’ and their perceived causes. ‘Triggers’ are thoughts or situations that lead to performance of the unwanted behavior. [↑](#footnote-ref-7)
8. Change or support change of the expectant woman’s physical or social surroundings to facilitate performing the behavior, create barriers to an unwanted behavior, or avoid cues to an unwanted behavior. [↑](#footnote-ref-8)
9. Demonstrate the behavior; provide an observable example of the behavior; draw attention to others’ performance of the behavior as a model. [↑](#footnote-ref-9)
10. Identify, introduce, or alter social or environmental prompts or cues to promote the wanted behavior. [↑](#footnote-ref-10)
11. Identify, alter, or remove social or environmental prompts or cues to deter the unwanted behavior. [↑](#footnote-ref-11)
12. Encourage the expectant woman to practice performing the behavior or substitute it for an unwanted behavior. [↑](#footnote-ref-12)
13. Encourage the expectant woman to compare the pros and cons of changing the behavior, or to compare the outcomes of changing versus not changing the behavior. Includes encouraging the expectant woman’s imagination or observation of either the consequences of the unwanted behavior or rewards for the wanted behavior [↑](#footnote-ref-13)
14. Present verbal or visual communication from a credible source in favor of or against the behavior. [↑](#footnote-ref-14)
15. Provide or arrange for the expectant woman to receive a *material incentive or reward* (something of value) or a *social incentive or reward* (words of congratulation), or removal of an unpleasant consequence for making progress in performing the behavior. Includes encouraging the expectant woman to use self-incentives or self-rewards. [↑](#footnote-ref-15)
16. Use a threat of future punishment or removal of a reward as a consequence of performance of an unwanted behavior; arrange for a negative consequence or punishment following performance of an unwanted behavior. [↑](#footnote-ref-16)
17. Suggest strategies to minimize demands on the expectant woman’s mental resources to make it easier for her to perform the behavior. [↑](#footnote-ref-17)
18. Suggest to the expectant woman that performing the behavior might serve as an example to others. [↑](#footnote-ref-18)
19. Assist the expectant woman to identify discrepancies between her behavior and her values or self-image; encourage her to self-identify as someone who *used to* perform the unwanted behavior; suggest her adopting a new perspective to change thoughts or emotions about the behavior. [↑](#footnote-ref-19)
20. Promote the expectant woman’s self-belief that she can successfully perform the behavior, for example by persuading her about her capabilities and encouraging her to mentally rehearse success, focus on past success or use positive self-talk. [↑](#footnote-ref-20)
21. Provide referral or information to link the expectant woman to a community resource to assist in performing the behavior; review progress in completing the referral; support connections in completing the referral or perform an interagency case review. [↑](#footnote-ref-21)
22. Review the expectant woman’s experience accessing community resources to help her perform the behavior; assist in overcoming barriers to completing a referral. [↑](#footnote-ref-22)
23. Ask about and act on the expectant woman’s ideas on how to assist her in adhering to guidance from other providers regarding performing the behavior. [↑](#footnote-ref-23)
24. Gather information about the expectant woman’s readiness to change the behavior. [↑](#footnote-ref-24)
25. Assist the expectant woman to: set a behavior change goal; develop a plan to meet the goal using strategies to overcome barriers and increase facilitators; review her progress toward the goal; modify the goal or plan as needed. [↑](#footnote-ref-25)
26. Monitor the expectant woman’s progress in changing the behavior; give feedback on that progress; establish ways for the expectant woman to self-monitor her progress. [↑](#footnote-ref-26)
27. Directly provide the expectant woman encouragement, emotional support or practical help to perform the behavior. [↑](#footnote-ref-27)
28. Suggest or assist the expectant woman to seek encouragement, emotional support or practical help to perform the behavior from a friend, relative, colleague, or group. [↑](#footnote-ref-28)
29. Provide written, verbal or visual information about the behavior’s health, emotional, social or environmental consequences; encourage her to assess her feelings after attempts to perform the behavior; raise her awareness of future regret about performing the unwanted behavior. [↑](#footnote-ref-29)
30. Provide information or instruction to shape the expectant woman’s knowledge of *how to perform the behavior*. This includes identification of behavioral ‘triggers’ and their perceived causes. ‘Triggers’ are thoughts or situations that lead to performance of the unwanted behavior. [↑](#footnote-ref-30)
31. Change or support change of the expectant woman’s physical or social surroundings to facilitate performing the behavior, create barriers to an unwanted behavior, or avoid cues to an unwanted behavior. [↑](#footnote-ref-31)
32. Demonstrate the behavior; provide an observable example of the behavior; draw attention to others’ performance of the behavior as a model. [↑](#footnote-ref-32)
33. Identify, introduce, or alter social or environmental prompts or cues to promote the wanted behavior. [↑](#footnote-ref-33)
34. Identify, alter, or remove social or environmental prompts or cues to deter the unwanted behavior. [↑](#footnote-ref-34)
35. Encourage the expectant woman to practice performing the behavior or substitute it for an unwanted behavior. [↑](#footnote-ref-35)
36. Encourage the expectant woman to compare the pros and cons of changing the behavior, or to compare the outcomes of changing versus not changing the behavior. Includes encouraging the expectant woman’s imagination or observation of either the consequences of the unwanted behavior or rewards for the wanted behavior [↑](#footnote-ref-36)
37. Present verbal or visual communication from a credible source in favor of or against the behavior. [↑](#footnote-ref-37)
38. Provide or arrange for the expectant woman to receive a *material incentive or reward* (something of value) or a *social incentive or reward* (words of congratulation), or removal of an unpleasant consequence for making progress in performing the behavior. Includes encouraging the expectant woman to use self-incentives or self-rewards. [↑](#footnote-ref-38)
39. Use a threat of future punishment or removal of a reward as a consequence of performance of an unwanted behavior; arrange for a negative consequence or punishment following performance of an unwanted behavior. [↑](#footnote-ref-39)
40. Suggest strategies to minimize demands on the expectant woman’s mental resources to make it easier for her to perform the behavior. [↑](#footnote-ref-40)
41. Suggest to the expectant woman that performing the behavior might serve as an example to others. [↑](#footnote-ref-41)
42. Assist the expectant woman to identify discrepancies between her behavior and her values or self-image; encourage her to self-identify as someone who *used to* perform the unwanted behavior; suggest her adopting a new perspective to change thoughts or emotions about the behavior. [↑](#footnote-ref-42)
43. Promote the expectant woman’s self-belief that she can successfully perform the behavior, for example by persuading her about her capabilities and encouraging her to mentally rehearse success, focus on past success or use positive self-talk. [↑](#footnote-ref-43)
44. Provide referral or information to link the expectant woman to a community resource to assist in performing the behavior; review progress in completing the referral; support connections in completing the referral or perform an interagency case review. [↑](#footnote-ref-44)
45. Review the expectant woman’s experience accessing community resources to help her perform the behavior; assist in overcoming barriers to completing a referral. [↑](#footnote-ref-45)
46. Ask about and act on the expectant woman’s ideas on how to assist her in adhering to guidance from other providers regarding performing the behavior. [↑](#footnote-ref-46)
47. Gather information about the expectant woman’s readiness to change the behavior. [↑](#footnote-ref-47)
48. Assist the expectant woman to: set a behavior change goal; develop a plan to meet the goal using strategies to overcome barriers and increase facilitators; review her progress toward the goal; modify the goal or plan as needed. [↑](#footnote-ref-48)
49. Monitor the expectant woman’s progress in changing the behavior; give feedback on that progress; establish ways for the expectant woman to self-monitor her progress. [↑](#footnote-ref-49)
50. Directly provide the expectant woman encouragement, emotional support or practical help to perform the behavior. [↑](#footnote-ref-50)
51. Suggest or assist the expectant woman to seek encouragement, emotional support or practical help to perform the behavior from a friend, relative, colleague, or group. [↑](#footnote-ref-51)
52. Provide written, verbal or visual information about the behavior’s health, emotional, social or environmental consequences; encourage her to assess her feelings after attempts to perform the behavior; raise her awareness of future regret about performing the unwanted behavior. [↑](#footnote-ref-52)
53. Provide information or instruction to shape the expectant woman’s knowledge of *how to perform the behavior*. This includes identification of behavioral ‘triggers’ and their perceived causes. ‘Triggers’ are thoughts or situations that lead to performance of the unwanted behavior. [↑](#footnote-ref-53)
54. Change or support change of the expectant woman’s physical or social surroundings to facilitate performing the behavior, create barriers to an unwanted behavior, or avoid cues to an unwanted behavior. [↑](#footnote-ref-54)
55. Demonstrate the behavior; provide an observable example of the behavior; draw attention to others’ performance of the behavior as a model. [↑](#footnote-ref-55)
56. Identify, introduce, or alter social or environmental prompts or cues to promote the wanted behavior. [↑](#footnote-ref-56)
57. Identify, alter, or remove social or environmental prompts or cues to deter the unwanted behavior. [↑](#footnote-ref-57)
58. Encourage the expectant woman to practice performing the behavior or substitute it for an unwanted behavior. [↑](#footnote-ref-58)
59. Encourage the expectant woman to compare the pros and cons of changing the behavior, or to compare the outcomes of changing versus not changing the behavior. Includes encouraging the expectant woman’s imagination or observation of either the consequences of the unwanted behavior or rewards for the wanted behavior [↑](#footnote-ref-59)
60. Present verbal or visual communication from a credible source in favor of or against the behavior. [↑](#footnote-ref-60)
61. Provide or arrange for the expectant woman to receive a *material incentive or reward* (something of value) or a *social incentive or reward* (words of congratulation), or removal of an unpleasant consequence for making progress in performing the behavior. Includes encouraging the expectant woman to use self-incentives or self-rewards. [↑](#footnote-ref-61)
62. Use a threat of future punishment or removal of a reward as a consequence of performance of an unwanted behavior; arrange for a negative consequence or punishment following performance of an unwanted behavior. [↑](#footnote-ref-62)
63. Suggest strategies to minimize demands on the expectant woman’s mental resources to make it easier for her to perform the behavior. [↑](#footnote-ref-63)
64. Suggest to the expectant woman that performing the behavior might serve as an example to others. [↑](#footnote-ref-64)
65. Assist the expectant woman to identify discrepancies between her behavior and her values or self-image; encourage her to self-identify as someone who *used to* perform the unwanted behavior; suggest her adopting a new perspective to change thoughts or emotions about the behavior. [↑](#footnote-ref-65)
66. Promote the expectant woman’s self-belief that she can successfully perform the behavior, for example by persuading her about her capabilities and encouraging her to mentally rehearse success, focus on past success or use positive self-talk. [↑](#footnote-ref-66)
67. Provide referral or information to link the expectant woman to a community resource to assist in performing the behavior; review progress in completing the referral; support connections in completing the referral or perform an interagency case review. [↑](#footnote-ref-67)
68. Review the expectant woman’s experience accessing community resources to help her perform the behavior; assist in overcoming barriers to completing a referral. [↑](#footnote-ref-68)
69. Ask about and act on the expectant woman’s ideas on how to assist her in adhering to guidance from other providers regarding performing the behavior. [↑](#footnote-ref-69)
70. Gather information about the expectant woman’s readiness to change the behavior. [↑](#footnote-ref-70)
71. Assist the expectant woman to: set a behavior change goal; develop a plan to meet the goal using strategies to overcome barriers and increase facilitators; review her progress toward the goal; modify the goal or plan as needed. [↑](#footnote-ref-71)
72. Monitor the expectant woman’s progress in changing the behavior; give feedback on that progress; establish ways for the expectant woman to self-monitor her progress. [↑](#footnote-ref-72)
73. Directly provide the expectant woman encouragement, emotional support or practical help to perform the behavior. [↑](#footnote-ref-73)
74. Suggest or assist the expectant woman to seek encouragement, emotional support or practical help to perform the behavior from a friend, relative, colleague, or group. [↑](#footnote-ref-74)
75. Provide written, verbal or visual information about the behavior’s health, emotional, social or environmental consequences; encourage her to assess her feelings after attempts to perform the behavior; raise her awareness of future regret about performing the unwanted behavior. [↑](#footnote-ref-75)
76. Provide information or instruction to shape the expectant woman’s knowledge of *how to perform the behavior*. This includes identification of behavioral ‘triggers’ and their perceived causes. ‘Triggers’ are thoughts or situations that lead to performance of the unwanted behavior. [↑](#footnote-ref-76)
77. Change or support change of the expectant woman’s physical or social surroundings to facilitate performing the behavior, create barriers to an unwanted behavior, or avoid cues to an unwanted behavior. [↑](#footnote-ref-77)
78. Demonstrate the behavior; provide an observable example of the behavior; draw attention to others’ performance of the behavior as a model. [↑](#footnote-ref-78)
79. Identify, introduce, or alter social or environmental prompts or cues to promote the wanted behavior. [↑](#footnote-ref-79)
80. Identify, alter, or remove social or environmental prompts or cues to deter the unwanted behavior. [↑](#footnote-ref-80)
81. Encourage the expectant woman to practice performing the behavior or substitute it for an unwanted behavior. [↑](#footnote-ref-81)
82. Encourage the expectant woman to compare the pros and cons of changing the behavior, or to compare the outcomes of changing versus not changing the behavior. Includes encouraging the expectant woman’s imagination or observation of either the consequences of the unwanted behavior or rewards for the wanted behavior [↑](#footnote-ref-82)
83. Present verbal or visual communication from a credible source in favor of or against the behavior. [↑](#footnote-ref-83)
84. Provide or arrange for the expectant woman to receive a *material incentive or reward* (something of value) or a *social incentive or reward* (words of congratulation), or removal of an unpleasant consequence for making progress in performing the behavior. Includes encouraging the expectant woman to use self-incentives or self-rewards. [↑](#footnote-ref-84)
85. Use a threat of future punishment or removal of a reward as a consequence of performance of an unwanted behavior; arrange for a negative consequence or punishment following performance of an unwanted behavior. [↑](#footnote-ref-85)
86. Suggest strategies to minimize demands on the expectant woman’s mental resources to make it easier for her to perform the behavior. [↑](#footnote-ref-86)
87. Suggest to the expectant woman that performing the behavior might serve as an example to others. [↑](#footnote-ref-87)
88. Assist the expectant woman to identify discrepancies between her behavior and her values or self-image; encourage her to self-identify as someone who *used to* perform the unwanted behavior; suggest her adopting a new perspective to change thoughts or emotions about the behavior. [↑](#footnote-ref-88)
89. Promote the expectant woman’s self-belief that she can successfully perform the behavior, for example by persuading her about her capabilities and encouraging her to mentally rehearse success, focus on past success or use positive self-talk. [↑](#footnote-ref-89)
90. Provide referral or information to link the expectant woman to a community resource to assist in performing the behavior; review progress in completing the referral; support connections in completing the referral or perform an interagency case review. [↑](#footnote-ref-90)
91. Review the expectant woman’s experience accessing community resources to help her perform the behavior; assist in overcoming barriers to completing a referral. [↑](#footnote-ref-91)
92. Ask about and act on the expectant woman’s ideas on how to assist her in adhering to guidance from other providers regarding performing the behavior. [↑](#footnote-ref-92)
93. Gather information about the expectant woman’s readiness to change the behavior. [↑](#footnote-ref-93)
94. Assist the expectant woman to: set a behavior change goal; develop a plan to meet the goal using strategies to overcome barriers and increase facilitators; review her progress toward the goal; modify the goal or plan as needed. [↑](#footnote-ref-94)
95. Monitor the expectant woman’s progress in changing the behavior; give feedback on that progress; establish ways for the expectant woman to self-monitor her progress. [↑](#footnote-ref-95)
96. Directly provide the expectant woman encouragement, emotional support or practical help to perform the behavior. [↑](#footnote-ref-96)
97. Suggest or assist the expectant woman to seek encouragement, emotional support or practical help to perform the behavior from a friend, relative, colleague, or group. [↑](#footnote-ref-97)
98. Provide written, verbal or visual information about the behavior’s health, emotional, social or environmental consequences; encourage her to assess her feelings after attempts to perform the behavior; raise her awareness of future regret about performing the unwanted behavior. [↑](#footnote-ref-98)
99. Provide information or instruction to shape the expectant woman’s knowledge of *how to perform the behavior*. This includes identification of behavioral ‘triggers’ and their perceived causes. ‘Triggers’ are thoughts or situations that lead to performance of the unwanted behavior. [↑](#footnote-ref-99)
100. Change or support change of the expectant woman’s physical or social surroundings to facilitate performing the behavior, create barriers to an unwanted behavior, or avoid cues to an unwanted behavior. [↑](#footnote-ref-100)
101. Demonstrate the behavior; provide an observable example of the behavior; draw attention to others’ performance of the behavior as a model. [↑](#footnote-ref-101)
102. Identify, introduce, or alter social or environmental prompts or cues to promote the wanted behavior. [↑](#footnote-ref-102)
103. Identify, alter, or remove social or environmental prompts or cues to deter the unwanted behavior. [↑](#footnote-ref-103)
104. Encourage the expectant woman to practice performing the behavior or substitute it for an unwanted behavior. [↑](#footnote-ref-104)
105. Encourage the expectant woman to compare the pros and cons of changing the behavior, or to compare the outcomes of changing versus not changing the behavior. Includes encouraging the expectant woman’s imagination or observation of either the consequences of the unwanted behavior or rewards for the wanted behavior [↑](#footnote-ref-105)
106. Present verbal or visual communication from a credible source in favor of or against the behavior. [↑](#footnote-ref-106)
107. Provide or arrange for the expectant woman to receive a *material incentive or reward* (something of value) or a *social incentive or reward* (words of congratulation), or removal of an unpleasant consequence for making progress in performing the behavior. Includes encouraging the expectant woman to use self-incentives or self-rewards. [↑](#footnote-ref-107)
108. Use a threat of future punishment or removal of a reward as a consequence of performance of an unwanted behavior; arrange for a negative consequence or punishment following performance of an unwanted behavior. [↑](#footnote-ref-108)
109. Suggest strategies to minimize demands on the expectant woman’s mental resources to make it easier for her to perform the behavior. [↑](#footnote-ref-109)
110. Suggest to the expectant woman that performing the behavior might serve as an example to others. [↑](#footnote-ref-110)
111. Assist the expectant woman to identify discrepancies between her behavior and her values or self-image; encourage her to self-identify as someone who *used to* perform the unwanted behavior; suggest her adopting a new perspective to change thoughts or emotions about the behavior. [↑](#footnote-ref-111)
112. Promote the expectant woman’s self-belief that she can successfully perform the behavior, for example by persuading her about her capabilities and encouraging her to mentally rehearse success, focus on past success or use positive self-talk. [↑](#footnote-ref-112)
113. Provide referral or information to link the expectant woman to a community resource to assist in performing the behavior; review progress in completing the referral; support connections in completing the referral or perform an interagency case review. [↑](#footnote-ref-113)
114. Review the expectant woman’s experience accessing community resources to help her perform the behavior; assist in overcoming barriers to completing a referral. [↑](#footnote-ref-114)
115. Ask about and act on the expectant woman’s ideas on how to assist her in adhering to guidance from other providers regarding performing the behavior. [↑](#footnote-ref-115)
116. Gather information about the expectant woman’s readiness to change the behavior. [↑](#footnote-ref-116)
117. Assist the expectant woman to: set a behavior change goal; develop a plan to meet the goal using strategies to overcome barriers and increase facilitators; review her progress toward the goal; modify the goal or plan as needed. [↑](#footnote-ref-117)
118. Monitor the expectant woman’s progress in changing the behavior; give feedback on that progress; establish ways for the expectant woman to self-monitor her progress. [↑](#footnote-ref-118)
119. Directly provide the expectant woman encouragement, emotional support or practical help to perform the behavior. [↑](#footnote-ref-119)
120. Suggest or assist the expectant woman to seek encouragement, emotional support or practical help to perform the behavior from a friend, relative, colleague, or group. [↑](#footnote-ref-120)
121. Provide written, verbal or visual information about the behavior’s health, emotional, social or environmental consequences; encourage her to assess her feelings after attempts to perform the behavior; raise her awareness of future regret about performing the unwanted behavior. [↑](#footnote-ref-121)
122. Provide information or instruction to shape the expectant woman’s knowledge of *how to perform the behavior*. This includes identification of behavioral ‘triggers’ and their perceived causes. ‘Triggers’ are thoughts or situations that lead to performance of the unwanted behavior. [↑](#footnote-ref-122)
123. Change or support change of the expectant woman’s physical or social surroundings to facilitate performing the behavior, create barriers to an unwanted behavior, or avoid cues to an unwanted behavior. [↑](#footnote-ref-123)
124. Demonstrate the behavior; provide an observable example of the behavior; draw attention to others’ performance of the behavior as a model. [↑](#footnote-ref-124)
125. Identify, introduce, or alter social or environmental prompts or cues to promote the wanted behavior. [↑](#footnote-ref-125)
126. Identify, alter, or remove social or environmental prompts or cues to deter the unwanted behavior. [↑](#footnote-ref-126)
127. Encourage the expectant woman to practice performing the behavior or substitute it for an unwanted behavior. [↑](#footnote-ref-127)
128. Encourage the expectant woman to compare the pros and cons of changing the behavior, or to compare the outcomes of changing versus not changing the behavior. Includes encouraging the expectant woman’s imagination or observation of either the consequences of the unwanted behavior or rewards for the wanted behavior [↑](#footnote-ref-128)
129. Present verbal or visual communication from a credible source in favor of or against the behavior. [↑](#footnote-ref-129)
130. Provide or arrange for the expectant woman to receive a *material incentive or reward* (something of value) or a *social incentive or reward* (words of congratulation), or removal of an unpleasant consequence for making progress in performing the behavior. Includes encouraging the expectant woman to use self-incentives or self-rewards. [↑](#footnote-ref-130)
131. Use a threat of future punishment or removal of a reward as a consequence of performance of an unwanted behavior; arrange for a negative consequence or punishment following performance of an unwanted behavior. [↑](#footnote-ref-131)
132. Suggest strategies to minimize demands on the expectant woman’s mental resources to make it easier for her to perform the behavior. [↑](#footnote-ref-132)
133. Suggest to the expectant woman that performing the behavior might serve as an example to others. [↑](#footnote-ref-133)
134. Assist the expectant woman to identify discrepancies between her behavior and her values or self-image; encourage her to self-identify as someone who *used to* perform the unwanted behavior; suggest her adopting a new perspective to change thoughts or emotions about the behavior. [↑](#footnote-ref-134)
135. Promote the expectant woman’s self-belief that she can successfully perform the behavior, for example by persuading her about her capabilities and encouraging her to mentally rehearse success, focus on past success or use positive self-talk. [↑](#footnote-ref-135)
136. Provide referral or information to link the expectant woman to a community resource to assist in performing the behavior; review progress in completing the referral; support connections in completing the referral or perform an interagency case review. [↑](#footnote-ref-136)
137. Review the expectant woman’s experience accessing community resources to help her perform the behavior; assist in overcoming barriers to completing a referral. [↑](#footnote-ref-137)
138. Ask about and act on the expectant woman’s ideas on how to assist her in adhering to guidance from other providers regarding performing the behavior. [↑](#footnote-ref-138)
139. ^1^ Gather information about the expectant woman’s readiness to change the behavior. [↑](#footnote-ref-139)
140. Assist the expectant woman to: set a behavior change goal; develop a plan to meet the goal using strategies to overcome barriers and increase facilitators; review her progress toward the goal; modify the goal or plan as needed. [↑](#footnote-ref-140)
141. Monitor the expectant woman’s progress in changing the behavior; give feedback on that progress; establish ways for the expectant woman to self-monitor her progress. [↑](#footnote-ref-141)
142. Directly provide the expectant woman encouragement, emotional support or practical help to perform the behavior. [↑](#footnote-ref-142)
143. Suggest or assist the expectant woman to seek encouragement, emotional support or practical help to perform the behavior from a friend, relative, colleague, or group. [↑](#footnote-ref-143)
144. Provide written, verbal or visual information about the behavior’s health, emotional, social or environmental consequences; encourage her to assess her feelings after attempts to perform the behavior; raise her awareness of future regret about performing the unwanted behavior. [↑](#footnote-ref-144)
145. Provide information or instruction to shape the expectant woman’s knowledge of *how to perform the behavior*. This includes identification of behavioral ‘triggers’ and their perceived causes. ‘Triggers’ are thoughts or situations that lead to performance of the unwanted behavior. [↑](#footnote-ref-145)
146. Change or support change of the expectant woman’s physical or social surroundings to facilitate performing the behavior, create barriers to an unwanted behavior, or avoid cues to an unwanted behavior. [↑](#footnote-ref-146)
147. Demonstrate the behavior; provide an observable example of the behavior; draw attention to others’ performance of the behavior as a model. [↑](#footnote-ref-147)
148. Identify, introduce, or alter social or environmental prompts or cues to promote the wanted behavior. [↑](#footnote-ref-148)
149. Identify, alter, or remove social or environmental prompts or cues to deter the unwanted behavior. [↑](#footnote-ref-149)
150. Encourage the expectant woman to practice performing the behavior or substitute it for an unwanted behavior. [↑](#footnote-ref-150)
151. Encourage the expectant woman to compare the pros and cons of changing the behavior, or to compare the outcomes of changing versus not changing the behavior. Includes encouraging the expectant woman’s imagination or observation of either the consequences of the unwanted behavior or rewards for the wanted behavior [↑](#footnote-ref-151)
152. Present verbal or visual communication from a credible source in favor of or against the behavior. [↑](#footnote-ref-152)
153. Provide or arrange for the expectant woman to receive a *material incentive or reward* (something of value) or a *social incentive or reward* (words of congratulation), or removal of an unpleasant consequence for making progress in performing the behavior. Includes encouraging the expectant woman to use self-incentives or self-rewards. [↑](#footnote-ref-153)
154. Use a threat of future punishment or removal of a reward as a consequence of performance of an unwanted behavior; arrange for a negative consequence or punishment following performance of an unwanted behavior. [↑](#footnote-ref-154)
155. Suggest strategies to minimize demands on the expectant woman’s mental resources to make it easier for her to perform the behavior. [↑](#footnote-ref-155)
156. Suggest to the expectant woman that performing the behavior might serve as an example to others. [↑](#footnote-ref-156)
157. Assist the expectant woman to identify discrepancies between her behavior and her values or self-image; encourage her to self-identify as someone who *used to* perform the unwanted behavior; suggest her adopting a new perspective to change thoughts or emotions about the behavior. [↑](#footnote-ref-157)
158. Promote the expectant woman’s self-belief that she can successfully perform the behavior, for example by persuading her about her capabilities and encouraging her to mentally rehearse success, focus on past success or use positive self-talk. [↑](#footnote-ref-158)
159. Provide referral or information to link the expectant woman to a community resource to assist in performing the behavior; review progress in completing the referral; support connections in completing the referral or perform an interagency case review. [↑](#footnote-ref-159)
160. Review the expectant woman’s experience accessing community resources to help her perform the behavior; assist in overcoming barriers to completing a referral. [↑](#footnote-ref-160)
161. Ask about and act on the expectant woman’s ideas on how to assist her in adhering to guidance from other providers regarding performing the behavior. [↑](#footnote-ref-161)
162. ^1^ Gather information about the expectant woman’s readiness to change the behavior. [↑](#footnote-ref-162)
163. Assist the expectant woman to: set a behavior change goal; develop a plan to meet the goal using strategies to overcome barriers and increase facilitators; review her progress toward the goal; modify the goal or plan as needed. [↑](#footnote-ref-163)
164. Monitor the expectant woman’s progress in changing the behavior; give feedback on that progress; establish ways for the expectant woman to self-monitor her progress. [↑](#footnote-ref-164)
165. Directly provide the expectant woman encouragement, emotional support or practical help to perform the behavior. [↑](#footnote-ref-165)
166. Suggest or assist the expectant woman to seek encouragement, emotional support or practical help to perform the behavior from a friend, relative, colleague, or group. [↑](#footnote-ref-166)
167. Provide written, verbal or visual information about the behavior’s health, emotional, social or environmental consequences; encourage her to assess her feelings after attempts to perform the behavior; raise her awareness of future regret about performing the unwanted behavior. [↑](#footnote-ref-167)
168. Provide information or instruction to shape the expectant woman’s knowledge of *how to perform the behavior*. This includes identification of behavioral ‘triggers’ and their perceived causes. ‘Triggers’ are thoughts or situations that lead to performance of the unwanted behavior. [↑](#footnote-ref-168)
169. Change or support change of the expectant woman’s physical or social surroundings to facilitate performing the behavior, create barriers to an unwanted behavior, or avoid cues to an unwanted behavior. [↑](#footnote-ref-169)
170. Demonstrate the behavior; provide an observable example of the behavior; draw attention to others’ performance of the behavior as a model. [↑](#footnote-ref-170)
171. Identify, introduce, or alter social or environmental prompts or cues to promote the wanted behavior. [↑](#footnote-ref-171)
172. Identify, alter, or remove social or environmental prompts or cues to deter the unwanted behavior. [↑](#footnote-ref-172)
173. Encourage the expectant woman to practice performing the behavior or substitute it for an unwanted behavior. [↑](#footnote-ref-173)
174. Encourage the expectant woman to compare the pros and cons of changing the behavior, or to compare the outcomes of changing versus not changing the behavior. Includes encouraging the expectant woman’s imagination or observation of either the consequences of the unwanted behavior or rewards for the wanted behavior [↑](#footnote-ref-174)
175. Present verbal or visual communication from a credible source in favor of or against the behavior. [↑](#footnote-ref-175)
176. Provide or arrange for the expectant woman to receive a *material incentive or reward* (something of value) or a *social incentive or reward* (words of congratulation), or removal of an unpleasant consequence for making progress in performing the behavior. Includes encouraging the expectant woman to use self-incentives or self-rewards. [↑](#footnote-ref-176)
177. Use a threat of future punishment or removal of a reward as a consequence of performance of an unwanted behavior; arrange for a negative consequence or punishment following performance of an unwanted behavior. [↑](#footnote-ref-177)
178. Suggest strategies to minimize demands on the expectant woman’s mental resources to make it easier for her to perform the behavior. [↑](#footnote-ref-178)
179. Suggest to the expectant woman that performing the behavior might serve as an example to others. [↑](#footnote-ref-179)
180. Assist the expectant woman to identify discrepancies between her behavior and her values or self-image; encourage her to self-identify as someone who *used to* perform the unwanted behavior; suggest her adopting a new perspective to change thoughts or emotions about the behavior. [↑](#footnote-ref-180)
181. Promote the expectant woman’s self-belief that she can successfully perform the behavior, for example by persuading her about her capabilities and encouraging her to mentally rehearse success, focus on past success or use positive self-talk. [↑](#footnote-ref-181)
182. Provide referral or information to link the expectant woman to a community resource to assist in performing the behavior; review progress in completing the referral; support connections in completing the referral or perform an interagency case review. [↑](#footnote-ref-182)
183. Review the expectant woman’s experience accessing community resources to help her perform the behavior; assist in overcoming barriers to completing a referral. [↑](#footnote-ref-183)
184. Ask about and act on the expectant woman’s ideas on how to assist her in adhering to guidance from other providers regarding performing the behavior. [↑](#footnote-ref-184)
185. Gather information about the expectant woman’s readiness to change the behavior. [↑](#footnote-ref-185)
186. Assist the expectant woman to: set a behavior change goal; develop a plan to meet the goal using strategies to overcome barriers and increase facilitators; review her progress toward the goal; modify the goal or plan as needed. [↑](#footnote-ref-186)
187. Monitor the expectant woman’s progress in changing the behavior; give feedback on that progress; establish ways for the expectant woman to self-monitor her progress. [↑](#footnote-ref-187)
188. Directly provide the expectant woman encouragement, emotional support or practical help to perform the behavior. [↑](#footnote-ref-188)
189. Suggest or assist the expectant woman to seek encouragement, emotional support or practical help to perform the behavior from a friend, relative, colleague, or group. [↑](#footnote-ref-189)
190. Provide written, verbal or visual information about the behavior’s health, emotional, social or environmental consequences; encourage her to assess her feelings after attempts to perform the behavior; raise her awareness of future regret about performing the unwanted behavior. [↑](#footnote-ref-190)
191. Provide information or instruction to shape the expectant woman’s knowledge of *how to perform the behavior*. This includes identification of behavioral ‘triggers’ and their perceived causes. ‘Triggers’ are thoughts or situations that lead to performance of the unwanted behavior. [↑](#footnote-ref-191)
192. Change or support change of the expectant woman’s physical or social surroundings to facilitate performing the behavior, create barriers to an unwanted behavior, or avoid cues to an unwanted behavior. [↑](#footnote-ref-192)
193. Demonstrate the behavior; provide an observable example of the behavior; draw attention to others’ performance of the behavior as a model. [↑](#footnote-ref-193)
194. Identify, introduce, or alter social or environmental prompts or cues to promote the wanted behavior. [↑](#footnote-ref-194)
195. Identify, alter, or remove social or environmental prompts or cues to deter the unwanted behavior. [↑](#footnote-ref-195)
196. Encourage the expectant woman to practice performing the behavior or substitute it for an unwanted behavior. [↑](#footnote-ref-196)
197. Encourage the expectant woman to compare the pros and cons of changing the behavior, or to compare the outcomes of changing versus not changing the behavior. Includes encouraging the expectant woman’s imagination or observation of either the consequences of the unwanted behavior or rewards for the wanted behavior [↑](#footnote-ref-197)
198. Present verbal or visual communication from a credible source in favor of or against the behavior. [↑](#footnote-ref-198)
199. Provide or arrange for the expectant woman to receive a *material incentive or reward* (something of value) or a *social incentive or reward* (words of congratulation), or removal of an unpleasant consequence for making progress in performing the behavior. Includes encouraging the expectant woman to use self-incentives or self-rewards. [↑](#footnote-ref-199)
200. Use a threat of future punishment or removal of a reward as a consequence of performance of an unwanted behavior; arrange for a negative consequence or punishment following performance of an unwanted behavior. [↑](#footnote-ref-200)
201. Suggest strategies to minimize demands on the expectant woman’s mental resources to make it easier for her to perform the behavior. [↑](#footnote-ref-201)
202. Suggest to the expectant woman that performing the behavior might serve as an example to others. [↑](#footnote-ref-202)
203. Assist the expectant woman to identify discrepancies between her behavior and her values or self-image; encourage her to self-identify as someone who *used to* perform the unwanted behavior; suggest her adopting a new perspective to change thoughts or emotions about the behavior. [↑](#footnote-ref-203)
204. Promote the expectant woman’s self-belief that she can successfully perform the behavior, for example by persuading her about her capabilities and encouraging her to mentally rehearse success, focus on past success or use positive self-talk. [↑](#footnote-ref-204)
205. Provide referral or information to link the expectant woman to a community resource to assist in performing the behavior; review progress in completing the referral; support connections in completing the referral or perform an interagency case review. [↑](#footnote-ref-205)
206. Review the expectant woman’s experience accessing community resources to help her perform the behavior; assist in overcoming barriers to completing a referral. [↑](#footnote-ref-206)
207. Ask about and act on the expectant woman’s ideas on how to assist her in adhering to guidance from other providers regarding performing the behavior. [↑](#footnote-ref-207)
208. Gather information about the expectant woman’s readiness to change the behavior. [↑](#footnote-ref-208)
209. Assist the expectant woman to: set a behavior change goal; develop a plan to meet the goal using strategies to overcome barriers and increase facilitators; review her progress toward the goal; modify the goal or plan as needed. [↑](#footnote-ref-209)
210. Monitor the expectant woman’s progress in changing the behavior; give feedback on that progress; establish ways for the expectant woman to self-monitor her progress. [↑](#footnote-ref-210)
211. Directly provide the expectant woman encouragement, emotional support or practical help to perform the behavior. [↑](#footnote-ref-211)
212. Suggest or assist the expectant woman to seek encouragement, emotional support or practical help to perform the behavior from a friend, relative, colleague, or group. [↑](#footnote-ref-212)
213. Provide written, verbal or visual information about the behavior’s health, emotional, social or environmental consequences; encourage her to assess her feelings after attempts to perform the behavior; raise her awareness of future regret about performing the unwanted behavior. [↑](#footnote-ref-213)
214. Provide information or instruction to shape the expectant woman’s knowledge of *how to perform the behavior*. This includes identification of behavioral ‘triggers’ and their perceived causes. ‘Triggers’ are thoughts or situations that lead to performance of the unwanted behavior. [↑](#footnote-ref-214)
215. Change or support change of the expectant woman’s physical or social surroundings to facilitate performing the behavior, create barriers to an unwanted behavior, or avoid cues to an unwanted behavior. [↑](#footnote-ref-215)
216. Demonstrate the behavior; provide an observable example of the behavior; draw attention to others’ performance of the behavior as a model. [↑](#footnote-ref-216)
217. Identify, introduce, or alter social or environmental prompts or cues to promote the wanted behavior. [↑](#footnote-ref-217)
218. Identify, alter, or remove social or environmental prompts or cues to deter the unwanted behavior. [↑](#footnote-ref-218)
219. Encourage the expectant woman to practice performing the behavior or substitute it for an unwanted behavior. [↑](#footnote-ref-219)
220. Encourage the expectant woman to compare the pros and cons of changing the behavior, or to compare the outcomes of changing versus not changing the behavior. Includes encouraging the expectant woman’s imagination or observation of either the consequences of the unwanted behavior or rewards for the wanted behavior [↑](#footnote-ref-220)
221. Present verbal or visual communication from a credible source in favor of or against the behavior. [↑](#footnote-ref-221)
222. Provide or arrange for the expectant woman to receive a *material incentive or reward* (something of value) or a *social incentive or reward* (words of congratulation), or removal of an unpleasant consequence for making progress in performing the behavior. Includes encouraging the expectant woman to use self-incentives or self-rewards. [↑](#footnote-ref-222)
223. Use a threat of future punishment or removal of a reward as a consequence of performance of an unwanted behavior; arrange for a negative consequence or punishment following performance of an unwanted behavior. [↑](#footnote-ref-223)
224. Suggest strategies to minimize demands on the expectant woman’s mental resources to make it easier for her to perform the behavior. [↑](#footnote-ref-224)
225. Suggest to the expectant woman that performing the behavior might serve as an example to others. [↑](#footnote-ref-225)
226. Assist the expectant woman to identify discrepancies between her behavior and her values or self-image; encourage her to self-identify as someone who *used to* perform the unwanted behavior; suggest her adopting a new perspective to change thoughts or emotions about the behavior. [↑](#footnote-ref-226)
227. Promote the expectant woman’s self-belief that she can successfully perform the behavior, for example by persuading her about her capabilities and encouraging her to mentally rehearse success, focus on past success or use positive self-talk. [↑](#footnote-ref-227)
228. Provide referral or information to link the expectant woman to a community resource to assist in performing the behavior; review progress in completing the referral; support connections in completing the referral or perform an interagency case review. [↑](#footnote-ref-228)
229. Review the expectant woman’s experience accessing community resources to help her perform the behavior; assist in overcoming barriers to completing a referral. [↑](#footnote-ref-229)
230. Ask about and act on the expectant woman’s ideas on how to assist her in adhering to guidance from other providers regarding performing the behavior. [↑](#footnote-ref-230)
231. Gather information about the expectant woman’s readiness to change the behavior. [↑](#footnote-ref-231)
232. Assist the expectant woman to: set a behavior change goal; develop a plan to meet the goal using strategies to overcome barriers and increase facilitators; review her progress toward the goal; modify the goal or plan as needed. [↑](#footnote-ref-232)
233. Monitor the expectant woman’s progress in changing the behavior; give feedback on that progress; establish ways for the expectant woman to self-monitor her progress. [↑](#footnote-ref-233)
234. Directly provide the expectant woman encouragement, emotional support or practical help to perform the behavior. [↑](#footnote-ref-234)
235. Suggest or assist the expectant woman to seek encouragement, emotional support or practical help to perform the behavior from a friend, relative, colleague, or group. [↑](#footnote-ref-235)
236. Provide written, verbal or visual information about the behavior’s health, emotional, social or environmental consequences; encourage her to assess her feelings after attempts to perform the behavior; raise her awareness of future regret about performing the unwanted behavior. [↑](#footnote-ref-236)
237. Provide information or instruction to shape the expectant woman’s knowledge of *how to perform the behavior*. This includes identification of behavioral ‘triggers’ and their perceived causes. ‘Triggers’ are thoughts or situations that lead to performance of the unwanted behavior. [↑](#footnote-ref-237)
238. Change or support change of the expectant woman’s physical or social surroundings to facilitate performing the behavior, create barriers to an unwanted behavior, or avoid cues to an unwanted behavior. [↑](#footnote-ref-238)
239. Demonstrate the behavior; provide an observable example of the behavior; draw attention to others’ performance of the behavior as a model. [↑](#footnote-ref-239)
240. Identify, introduce, or alter social or environmental prompts or cues to promote the wanted behavior. [↑](#footnote-ref-240)
241. Identify, alter, or remove social or environmental prompts or cues to deter the unwanted behavior. [↑](#footnote-ref-241)
242. Encourage the expectant woman to practice performing the behavior or substitute it for an unwanted behavior. [↑](#footnote-ref-242)
243. Encourage the expectant woman to compare the pros and cons of changing the behavior, or to compare the outcomes of changing versus not changing the behavior. Includes encouraging the expectant woman’s imagination or observation of either the consequences of the unwanted behavior or rewards for the wanted behavior [↑](#footnote-ref-243)
244. Present verbal or visual communication from a credible source in favor of or against the behavior. [↑](#footnote-ref-244)
245. Provide or arrange for the expectant woman to receive a *material incentive or reward* (something of value) or a *social incentive or reward* (words of congratulation), or removal of an unpleasant consequence for making progress in performing the behavior. Includes encouraging the expectant woman to use self-incentives or self-rewards. [↑](#footnote-ref-245)
246. Use a threat of future punishment or removal of a reward as a consequence of performance of an unwanted behavior; arrange for a negative consequence or punishment following performance of an unwanted behavior. [↑](#footnote-ref-246)
247. Suggest strategies to minimize demands on the expectant woman’s mental resources to make it easier for her to perform the behavior. [↑](#footnote-ref-247)
248. Suggest to the expectant woman that performing the behavior might serve as an example to others. [↑](#footnote-ref-248)
249. Assist the expectant woman to identify discrepancies between her behavior and her values or self-image; encourage her to self-identify as someone who *used to* perform the unwanted behavior; suggest her adopting a new perspective to change thoughts or emotions about the behavior. [↑](#footnote-ref-249)
250. Promote the expectant woman’s self-belief that she can successfully perform the behavior, for example by persuading her about her capabilities and encouraging her to mentally rehearse success, focus on past success or use positive self-talk. [↑](#footnote-ref-250)
251. Provide referral or information to link the expectant woman to a community resource to assist in performing the behavior; review progress in completing the referral; support connections in completing the referral or perform an interagency case review. [↑](#footnote-ref-251)
252. Review the expectant woman’s experience accessing community resources to help her perform the behavior; assist in overcoming barriers to completing a referral. [↑](#footnote-ref-252)
253. Ask about and act on the expectant woman’s ideas on how to assist her in adhering to guidance from other providers regarding performing the behavior. [↑](#footnote-ref-253)
254. Gather information about the expectant woman’s readiness to change the behavior. [↑](#footnote-ref-254)
255. Assist the expectant woman to: set a behavior change goal; develop a plan to meet the goal using strategies to overcome barriers and increase facilitators; review her progress toward the goal; modify the goal or plan as needed. [↑](#footnote-ref-255)
256. Monitor the expectant woman’s progress in changing the behavior; give feedback on that progress; establish ways for the expectant woman to self-monitor her progress. [↑](#footnote-ref-256)
257. Directly provide the expectant woman encouragement, emotional support or practical help to perform the behavior. [↑](#footnote-ref-257)
258. Suggest or assist the expectant woman to seek encouragement, emotional support or practical help to perform the behavior from a friend, relative, colleague, or group. [↑](#footnote-ref-258)
259. Provide written, verbal or visual information about the behavior’s health, emotional, social or environmental consequences; encourage her to assess her feelings after attempts to perform the behavior; raise her awareness of future regret about performing the unwanted behavior. [↑](#footnote-ref-259)
260. Provide information or instruction to shape the expectant woman’s knowledge of *how to perform the behavior*. This includes identification of behavioral ‘triggers’ and their perceived causes. ‘Triggers’ are thoughts or situations that lead to performance of the unwanted behavior. [↑](#footnote-ref-260)
261. Change or support change of the expectant woman’s physical or social surroundings to facilitate performing the behavior, create barriers to an unwanted behavior, or avoid cues to an unwanted behavior. [↑](#footnote-ref-261)
262. Demonstrate the behavior; provide an observable example of the behavior; draw attention to others’ performance of the behavior as a model. [↑](#footnote-ref-262)
263. Identify, introduce, or alter social or environmental prompts or cues to promote the wanted behavior. [↑](#footnote-ref-263)
264. Identify, alter, or remove social or environmental prompts or cues to deter the unwanted behavior. [↑](#footnote-ref-264)
265. Encourage the expectant woman to practice performing the behavior or substitute it for an unwanted behavior. [↑](#footnote-ref-265)
266. Encourage the expectant woman to compare the pros and cons of changing the behavior, or to compare the outcomes of changing versus not changing the behavior. Includes encouraging the expectant woman’s imagination or observation of either the consequences of the unwanted behavior or rewards for the wanted behavior [↑](#footnote-ref-266)
267. Present verbal or visual communication from a credible source in favor of or against the behavior. [↑](#footnote-ref-267)
268. Provide or arrange for the expectant woman to receive a *material incentive or reward* (something of value) or a *social incentive or reward* (words of congratulation), or removal of an unpleasant consequence for making progress in performing the behavior. Includes encouraging the expectant woman to use self-incentives or self-rewards. [↑](#footnote-ref-268)
269. Use a threat of future punishment or removal of a reward as a consequence of performance of an unwanted behavior; arrange for a negative consequence or punishment following performance of an unwanted behavior. [↑](#footnote-ref-269)
270. Suggest strategies to minimize demands on the expectant woman’s mental resources to make it easier for her to perform the behavior. [↑](#footnote-ref-270)
271. Suggest to the expectant woman that performing the behavior might serve as an example to others. [↑](#footnote-ref-271)
272. Assist the expectant woman to identify discrepancies between her behavior and her values or self-image; encourage her to self-identify as someone who *used to* perform the unwanted behavior; suggest her adopting a new perspective to change thoughts or emotions about the behavior. [↑](#footnote-ref-272)
273. Promote the expectant woman’s self-belief that she can successfully perform the behavior, for example by persuading her about her capabilities and encouraging her to mentally rehearse success, focus on past success or use positive self-talk. [↑](#footnote-ref-273)
274. Provide referral or information to link the expectant woman to a community resource to assist in performing the behavior; review progress in completing the referral; support connections in completing the referral or perform an interagency case review. [↑](#footnote-ref-274)
275. Review the expectant woman’s experience accessing community resources to help her perform the behavior; assist in overcoming barriers to completing a referral. [↑](#footnote-ref-275)
276. Ask about and act on the expectant woman’s ideas on how to assist her in adhering to guidance from other providers regarding performing the behavior. [↑](#footnote-ref-276)
277. Gather information about the expectant woman’s readiness to change the behavior. [↑](#footnote-ref-277)
278. Assist the expectant woman to: set a behavior change goal; develop a plan to meet the goal using strategies to overcome barriers and increase facilitators; review her progress toward the goal; modify the goal or plan as needed. [↑](#footnote-ref-278)
279. Monitor the expectant woman’s progress in changing the behavior; give feedback on that progress; establish ways for the expectant woman to self-monitor her progress. [↑](#footnote-ref-279)
280. Directly provide the expectant woman encouragement, emotional support or practical help to perform the behavior. [↑](#footnote-ref-280)
281. Suggest or assist the expectant woman to seek encouragement, emotional support or practical help to perform the behavior from a friend, relative, colleague, or group. [↑](#footnote-ref-281)
282. Provide written, verbal or visual information about the behavior’s health, emotional, social or environmental consequences; encourage her to assess her feelings after attempts to perform the behavior; raise her awareness of future regret about performing the unwanted behavior. [↑](#footnote-ref-282)
283. Provide information or instruction to shape the expectant woman’s knowledge of *how to perform the behavior*. This includes identification of behavioral ‘triggers’ and their perceived causes. ‘Triggers’ are thoughts or situations that lead to performance of the unwanted behavior. [↑](#footnote-ref-283)
284. Change or support change of the expectant woman’s physical or social surroundings to facilitate performing the behavior, create barriers to an unwanted behavior, or avoid cues to an unwanted behavior. [↑](#footnote-ref-284)
285. Demonstrate the behavior; provide an observable example of the behavior; draw attention to others’ performance of the behavior as a model. [↑](#footnote-ref-285)
286. Identify, introduce, or alter social or environmental prompts or cues to promote the wanted behavior. [↑](#footnote-ref-286)
287. Identify, alter, or remove social or environmental prompts or cues to deter the unwanted behavior. [↑](#footnote-ref-287)
288. Encourage the expectant woman to practice performing the behavior or substitute it for an unwanted behavior. [↑](#footnote-ref-288)
289. Encourage the expectant woman to compare the pros and cons of changing the behavior, or to compare the outcomes of changing versus not changing the behavior. Includes encouraging the expectant woman’s imagination or observation of either the consequences of the unwanted behavior or rewards for the wanted behavior [↑](#footnote-ref-289)
290. Present verbal or visual communication from a credible source in favor of or against the behavior. [↑](#footnote-ref-290)
291. Provide or arrange for the expectant woman to receive a *material incentive or reward* (something of value) or a *social incentive or reward* (words of congratulation), or removal of an unpleasant consequence for making progress in performing the behavior. Includes encouraging the expectant woman to use self-incentives or self-rewards. [↑](#footnote-ref-291)
292. Use a threat of future punishment or removal of a reward as a consequence of performance of an unwanted behavior; arrange for a negative consequence or punishment following performance of an unwanted behavior. [↑](#footnote-ref-292)
293. Suggest strategies to minimize demands on the expectant woman’s mental resources to make it easier for her to perform the behavior. [↑](#footnote-ref-293)
294. Suggest to the expectant woman that performing the behavior might serve as an example to others. [↑](#footnote-ref-294)
295. Assist the expectant woman to identify discrepancies between her behavior and her values or self-image; encourage her to self-identify as someone who *used to* perform the unwanted behavior; suggest her adopting a new perspective to change thoughts or emotions about the behavior. [↑](#footnote-ref-295)
296. Promote the expectant woman’s self-belief that she can successfully perform the behavior, for example by persuading her about her capabilities and encouraging her to mentally rehearse success, focus on past success or use positive self-talk. [↑](#footnote-ref-296)
297. Provide referral or information to link the expectant woman to a community resource to assist in performing the behavior; review progress in completing the referral; support connections in completing the referral or perform an interagency case review. [↑](#footnote-ref-297)
298. Review the expectant woman’s experience accessing community resources to help her perform the behavior; assist in overcoming barriers to completing a referral. [↑](#footnote-ref-298)
299. Ask about and act on the expectant woman’s ideas on how to assist her in adhering to guidance from other providers regarding performing the behavior. [↑](#footnote-ref-299)
300. Gather information about the expectant woman’s readiness to change the behavior. [↑](#footnote-ref-300)
301. Assist the expectant woman to: set a behavior change goal; develop a plan to meet the goal using strategies to overcome barriers and increase facilitators; review her progress toward the goal; modify the goal or plan as needed. [↑](#footnote-ref-301)
302. Monitor the expectant woman’s progress in changing the behavior; give feedback on that progress; establish ways for the expectant woman to self-monitor her progress. [↑](#footnote-ref-302)
303. Directly provide the expectant woman encouragement, emotional support or practical help to perform the behavior. [↑](#footnote-ref-303)
304. Suggest or assist the expectant woman to seek encouragement, emotional support or practical help to perform the behavior from a friend, relative, colleague, or group. [↑](#footnote-ref-304)
305. Provide written, verbal or visual information about the behavior’s health, emotional, social or environmental consequences; encourage her to assess her feelings after attempts to perform the behavior; raise her awareness of future regret about performing the unwanted behavior. [↑](#footnote-ref-305)
306. Provide information or instruction to shape the expectant woman’s knowledge of *how to perform the behavior*. This includes identification of behavioral ‘triggers’ and their perceived causes. ‘Triggers’ are thoughts or situations that lead to performance of the unwanted behavior. [↑](#footnote-ref-306)
307. Change or support change of the expectant woman’s physical or social surroundings to facilitate performing the behavior, create barriers to an unwanted behavior, or avoid cues to an unwanted behavior. [↑](#footnote-ref-307)
308. Demonstrate the behavior; provide an observable example of the behavior; draw attention to others’ performance of the behavior as a model. [↑](#footnote-ref-308)
309. Identify, introduce, or alter social or environmental prompts or cues to promote the wanted behavior. [↑](#footnote-ref-309)
310. Identify, alter, or remove social or environmental prompts or cues to deter the unwanted behavior. [↑](#footnote-ref-310)
311. Encourage the expectant woman to practice performing the behavior or substitute it for an unwanted behavior. [↑](#footnote-ref-311)
312. Encourage the expectant woman to compare the pros and cons of changing the behavior, or to compare the outcomes of changing versus not changing the behavior. Includes encouraging the expectant woman’s imagination or observation of either the consequences of the unwanted behavior or rewards for the wanted behavior [↑](#footnote-ref-312)
313. Present verbal or visual communication from a credible source in favor of or against the behavior. [↑](#footnote-ref-313)
314. Provide or arrange for the expectant woman to receive a *material incentive or reward* (something of value) or a *social incentive or reward* (words of congratulation), or removal of an unpleasant consequence for making progress in performing the behavior. Includes encouraging the expectant woman to use self-incentives or self-rewards. [↑](#footnote-ref-314)
315. Use a threat of future punishment or removal of a reward as a consequence of performance of an unwanted behavior; arrange for a negative consequence or punishment following performance of an unwanted behavior. [↑](#footnote-ref-315)
316. Suggest strategies to minimize demands on the expectant woman’s mental resources to make it easier for her to perform the behavior. [↑](#footnote-ref-316)
317. Suggest to the expectant woman that performing the behavior might serve as an example to others. [↑](#footnote-ref-317)
318. Assist the expectant woman to identify discrepancies between her behavior and her values or self-image; encourage her to self-identify as someone who *used to* perform the unwanted behavior; suggest her adopting a new perspective to change thoughts or emotions about the behavior. [↑](#footnote-ref-318)
319. Promote the expectant woman’s self-belief that she can successfully perform the behavior, for example by persuading her about her capabilities and encouraging her to mentally rehearse success, focus on past success or use positive self-talk. [↑](#footnote-ref-319)
320. Provide referral or information to link the expectant woman to a community resource to assist in performing the behavior; review progress in completing the referral; support connections in completing the referral or perform an interagency case review. [↑](#footnote-ref-320)
321. Review the expectant woman’s experience accessing community resources to help her perform the behavior; assist in overcoming barriers to completing a referral. [↑](#footnote-ref-321)
322. Ask about and act on the expectant woman’s ideas on how to assist her in adhering to guidance from other providers regarding performing the behavior. [↑](#footnote-ref-322)
