# Supplementary material for: Changing the home visiting research paradigm: models’ perspectives on behavioral pathways and intervention techniques to promote good birth outcomes
Source: BMC Public Health. 2022 May 21;22:1024. doi: 10.1186/s12889-022-13010-5 (PMC9123293; doi:10.1186/s12889-022-13010-5)
Supplement: Supplementary file 3 — Additional file 3. [file 12889_2022_13010_MOESM3_ESM.docx]

**Cross-Model Precision Prenatal HV Project Model Survey 3**

**Overview**

Thank you for completing Surveys 1 and 2.

The purpose of Survey 3 is to learn how your model views the relative emphasis home visitors should give to using specific techniques to promote behaviors to reduce each of the risk factors.

Each model’s version of Survey 3 is tailored to its responses in Surveys 1 and 2.

Your version of Survey 3 focuses on four behaviors: physical activity, adherence to prenatal care schedule, engagement in stress reduction activities, and use of social support.

There is one table for each behavior.

Each table lists only the techniques your model rated in Survey 2 as required or recommended for promoting the behavior.

**INSTRUCTIONS FOR COMPLETING EACH TABLE**

Rate your model’s expectations for the ***relative emphasis*** home visitors should give to using each technique to promote the behavior that is the focus of the table.

Your model might have exactly the same expectation for emphasis in using a particular technique across all risk factors, or it might have different expectations, depending on the risk factor. Each table will allow us to learn about that.

Response choices are 1 (low emphasis) to 5 (high emphasis) and “no stance” if your model has no expectation regarding emphasis in using a particular technique. Your model might have expectations regarding some techniques but not others. Each table will allow us to learn about that.

Note: Some techniques include multiple strategies, as defined in the table footnotes.  If your model expects different levels of emphasis in using strategies within a technique, select the response that reflects the highest expected level of emphasis among the strategies.

**The following represents the full set of possible questions in Survey 3. Survey 3 was customized according to each model based on their responses to surveys 1 and 2 so that only priority risks (low, moderate, or high), endorsed behaviors (required or recommended), and endorsed techniques (required or recommended) were included.*

**A**. **Techniques to Promote Engaging in Physical Activity**

| Rate your model’s expectations for home visitors’ *relative emphasis* in using each technique to promote expectant women’s **engagement in physical activity**.  Note: If your model expects different levels of emphasis in using strategies within a technique, select the response that reflects the highest expected level of emphasis among the strategies. | | | | |
| --- | --- | --- | --- | --- |
|  | **Risk Factors for Low Birthweight and Preterm Birth** | | | |
| **Technique** | **High Blood Pressure** | **Diabetes** | **High Stress** | **Depression** |
| Assess readiness for change^[[1]](#footnote-1)^ | Select response | Select response | Select response | Select response |
| Goals & planning^[[2]](#footnote-2)^ | Select response | Select response | Select response | Select response |
| Monitoring & feedback^[[3]](#footnote-3)^ | Select response | Select response | Select response | Select response |
| Provide social support^[[4]](#footnote-4)^ | Select response | Select response | Select response | Select response |
| Suggest or arrange social support^[[5]](#footnote-5)^ | Select response | Select response | Select response | Select response |
| Natural consequences^[[6]](#footnote-6)^ | Select response | Select response | Select response | Select response |
| Shape knowledge of behavior^[[7]](#footnote-7)^ | Select response | Select response | Select response | Select response |
| Antecedents^[[8]](#footnote-8)^ | Select response | Select response | Select response | Select response |
| Behavior observation^[[9]](#footnote-9)^ | Select response | Select response | Select response | Select response |
| Associations to promote wanted behavior^[[10]](#footnote-10)^ | Select response | Select response | Select response | Select response |
| Associations to deter unwanted behavior^[[11]](#footnote-11)^ | Select response | Select response | Select response | Select response |
| Repetition & substitution^[[12]](#footnote-12)^ | Select response | Select response | Select response | Select response |
| Comparison of outcomes^[[13]](#footnote-13)^ | Select response | Select response | Select response | Select response |
| Credible source^[[14]](#footnote-14)^ | Select response | Select response | Select response | Select response |
| Incentives & rewards^[[15]](#footnote-15)^ | Select response | Select response | Select response | Select response |
| Scheduled consequences^[[16]](#footnote-16)^ | Select response | Select response | Select response | Select response |
| Mental regulation^[[17]](#footnote-17)^ | Select response | Select response | Select response | Select response |
| Identity as example to others^[[18]](#footnote-18)^ | Select response | Select response | Select response | Select response |
| Self-identity^[[19]](#footnote-19)^ | Select response | Select response | Select response | Select response |
| Self-belief^[[20]](#footnote-20)^ | Select response | Select response | Select response | Select response |
| Referral & linkage^[[21]](#footnote-21)^ | Select response | Select response | Select response | Select response |
| Monitoring & follow-up of referral^[[22]](#footnote-22)^ | Select response | Select response | Select response | Select response |
| Coordination with other services^[[23]](#footnote-23)^ | Select response | Select response | Select response | Select response |

**B. Techniques to Promote Adherence to Prenatal Care Visit Schedule**

| Rate your model’s expectations for home visitors’ *relative emphasis* in using each technique to promote expectant women’s **adherence to prenatal care visit schedule**.  Note: If your model expects different levels of emphasis in using strategies within a technique, select the response that reflects the highest expected level of emphasis among the strategies. | | | | |
| --- | --- | --- | --- | --- |
|  | **Risk Factors for Low Birthweight and Preterm Birth** | | | |
| **Technique** | **High Blood Pressure** | **Diabetes** | **Depression** | **Inadequate Prenatal Care** |
| Assess readiness for change^[[24]](#footnote-24)^ | Select response | Select response | Select response | Select response |
| Goals & planning^[[25]](#footnote-25)^ | Select response | Select response | Select response | Select response |
| Monitoring & feedback^[[26]](#footnote-26)^ | Select response | Select response | Select response | Select response |
| Provide social support^[[27]](#footnote-27)^ | Select response | Select response | Select response | Select response |
| Suggest or arrange social support^[[28]](#footnote-28)^ | Select response | Select response | Select response | Select response |
| Natural consequences^[[29]](#footnote-29)^ | Select response | Select response | Select response | Select response |
| Shape knowledge of behavior^[[30]](#footnote-30)^ | Select response | Select response | Select response | Select response |
| Antecedents^[[31]](#footnote-31)^ | Select response | Select response | Select response | Select response |
| Behavior observation^[[32]](#footnote-32)^ | Select response | Select response | Select response | Select response |
| Associations to promote wanted behavior^[[33]](#footnote-33)^ | Select response | Select response | Select response | Select response |
| Associations to deter unwanted behavior^[[34]](#footnote-34)^ | Select response | Select response | Select response | Select response |
| Repetition & substitution^[[35]](#footnote-35)^ | Select response | Select response | Select response | Select response |
| Comparison of outcomes^[[36]](#footnote-36)^ | Select response | Select response | Select response | Select response |
| Credible source^[[37]](#footnote-37)^ | Select response | Select response | Select response | Select response |
| Incentives & rewards^[[38]](#footnote-38)^ | Select response | Select response | Select response | Select response |
| Scheduled consequences^[[39]](#footnote-39)^ | Select response | Select response | Select response | Select response |
| Mental regulation^[[40]](#footnote-40)^ | Select response | Select response | Select response | Select response |
| Identity as example to others^[[41]](#footnote-41)^ | Select response | Select response | Select response | Select response |
| Self-identity^[[42]](#footnote-42)^ | Select response | Select response | Select response | Select response |
| Self-belief^[[43]](#footnote-43)^ | Select response | Select response | Select response | Select response |
| Referral & linkage^[[44]](#footnote-44)^ | Select response | Select response | Select response | Select response |
| Monitoring & follow-up of referral^[[45]](#footnote-45)^ | Select response | Select response | Select response | Select response |
| Coordination with other services^[[46]](#footnote-46)^ | Select response | Select response | Select response | Select response |

**C. Techniques to Promote Engaging in Stress Reduction Activities**

| Rate your model’s expectations for home visitors’ *relative emphasis* in using each technique to promote expectant women’s **engagement in stress reduction activities**.  Note: If your model expects different levels of emphasis in using strategies within a technique, select the response that reflects the highest expected level of emphasis among the strategies. | | | | | | |
| --- | --- | --- | --- | --- | --- | --- |
|  | **Risk Factors for Low Birthweight and Preterm Birth** | | | | | |
| **Technique** | **IPV** | **High Stress** | **Depression** | **Tobacco Use** | **Alcohol Use** | **Illicit Drug Use** |
| Assess readiness for change^[[47]](#footnote-47)^ | Select response | Select response | Select response | Select response | Select response | Select response |
| Goals & planning^[[48]](#footnote-48)^ | Select response | Select response | Select response | Select response | Select response | Select response |
| Monitoring & feedback^[[49]](#footnote-49)^ | Select response | Select response | Select response | Select response | Select response | Select response |
| Provide social support^[[50]](#footnote-50)^ | Select response | Select response | Select response | Select response | Select response | Select response |
| Suggest or arrange social support^[[51]](#footnote-51)^ | Select response | Select response | Select response | Select response | Select response | Select response |
| Natural consequences^[[52]](#footnote-52)^ | Select response | Select response | Select response | Select response | Select response | Select response |
| Shape knowledge of behavior^[[53]](#footnote-53)^ | Select response | Select response | Select response | Select response | Select response | Select response |
| Antecedents^[[54]](#footnote-54)^ | Select response | Select response | Select response | Select response | Select response | Select response |
| Behavior observation^[[55]](#footnote-55)^ | Select response | Select response | Select response | Select response | Select response | Select response |
| Associations to promote wanted behavior^[[56]](#footnote-56)^ | Select response | Select response | Select response | Select response | Select response | Select response |
| Associations to deter unwanted behavior^[[57]](#footnote-57)^ | Select response | Select response | Select response | Select response | Select response | Select response |
| Repetition & substitution^[[58]](#footnote-58)^ | Select response | Select response | Select response | Select response | Select response | Select response |
| Comparison of outcomes^[[59]](#footnote-59)^ | Select response | Select response | Select response | Select response | Select response | Select response |
| Credible source^[[60]](#footnote-60)^ | Select response | Select response | Select response | Select response | Select response | Select response |
| Incentives & rewards^[[61]](#footnote-61)^ | Select response | Select response | Select response | Select response | Select response | Select response |
| Scheduled consequences^[[62]](#footnote-62)^ | Select response | Select response | Select response | Select response | Select response | Select response |
| Mental regulation^[[63]](#footnote-63)^ | Select response | Select response | Select response | Select response | Select response | Select response |
| Identity as example to others^[[64]](#footnote-64)^ | Select response | Select response | Select response | Select response | Select response | Select response |
| Self-identity^[[65]](#footnote-65)^ | Select response | Select response | Select response | Select response | Select response | Select response |
| Self-belief^[[66]](#footnote-66)^ | Select response | Select response | Select response | Select response | Select response | Select response |
| Referral & linkage^[[67]](#footnote-67)^ | Select response | Select response | Select response | Select response | Select response | Select response |
| Monitoring & follow-up of referral^[[68]](#footnote-68)^ | Select response | Select response | Select response | Select response | Select response | Select response |
| Coordination with other services^[[69]](#footnote-69)^ | Select response | Select response | Select response | Select response | Select response | Select response |

**D. Techniques to Promote Social Support Use**

| Rate your model’s expectations for home visitors’ *relative emphasis* in using each technique to promote expectant women’s **use of social supports**.  Note: If your model expects different levels of emphasis in using strategies within a technique, select the response that reflects the highest expected level of emphasis among the strategies. | | | | | | |
| --- | --- | --- | --- | --- | --- | --- |
|  | **Risk Factors for Low Birthweight and Preterm Birth** | | | | | |
| **Technique** | **IPV** | **High Stress** | **Depression** | **Tobacco Use** | **Alcohol Use** | **Illicit Drug Use** |
| Assess readiness for change^[[70]](#footnote-70)^ | Select response | Select response | Select response | Select response | Select response | Select response |
| Goals & planning^[[71]](#footnote-71)^ | Select response | Select response | Select response | Select response | Select response | Select response |
| Monitoring & feedback^[[72]](#footnote-72)^ | Select response | Select response | Select response | Select response | Select response | Select response |
| Provide social support^[[73]](#footnote-73)^ | Select response | Select response | Select response | Select response | Select response | Select response |
| Suggest or arrange social support^[[74]](#footnote-74)^ | Select response | Select response | Select response | Select response | Select response | Select response |
| Natural consequences^[[75]](#footnote-75)^ | Select response | Select response | Select response | Select response | Select response | Select response |
| Shape knowledge of behavior^[[76]](#footnote-76)^ | Select response | Select response | Select response | Select response | Select response | Select response |
| Antecedents^[[77]](#footnote-77)^ | Select response | Select response | Select response | Select response | Select response | Select response |
| Behavior observation^[[78]](#footnote-78)^ | Select response | Select response | Select response | Select response | Select response | Select response |
| Associations to promote wanted behavior^[[79]](#footnote-79)^ | Select response | Select response | Select response | Select response | Select response | Select response |
| Associations to deter unwanted behavior^[[80]](#footnote-80)^ | Select response | Select response | Select response | Select response | Select response | Select response |
| Repetition & substitution^[[81]](#footnote-81)^ | Select response | Select response | Select response | Select response | Select response | Select response |
| Comparison of outcomes^[[82]](#footnote-82)^ | Select response | Select response | Select response | Select response | Select response | Select response |
| Credible source^[[83]](#footnote-83)^ | Select response | Select response | Select response | Select response | Select response | Select response |
| Incentives & rewards^[[84]](#footnote-84)^ | Select response | Select response | Select response | Select response | Select response | Select response |
| Scheduled consequences^[[85]](#footnote-85)^ | Select response | Select response | Select response | Select response | Select response | Select response |
| Mental regulation^[[86]](#footnote-86)^ | Select response | Select response | Select response | Select response | Select response | Select response |
| Identity as example to others^[[87]](#footnote-87)^ | Select response | Select response | Select response | Select response | Select response | Select response |
| Self-identity^[[88]](#footnote-88)^ | Select response | Select response | Select response | Select response | Select response | Select response |
| Self-belief^[[89]](#footnote-89)^ | Select response | Select response | Select response | Select response | Select response | Select response |
| Referral & linkage^[[90]](#footnote-90)^ | Select response | Select response | Select response | Select response | Select response | Select response |
| Monitoring & follow-up of referral^[[91]](#footnote-91)^ | Select response | Select response | Select response | Select response | Select response | Select response |
| Coordination with other services^[[92]](#footnote-92)^ | Select response | Select response | Select response | Select response | Select response | Select response |

1. Gather information about the expectant woman’s readiness to change the behavior. [↑](#footnote-ref-1)
2. Assist the expectant woman to: set a behavior change goal; develop a plan to meet the goal using strategies to overcome barriers and increase facilitators; review her progress toward the goal; modify the goal or plan as needed. [↑](#footnote-ref-2)
3. Monitor the expectant woman’s progress in changing the behavior; give feedback on that progress; establish ways for the expectant woman to self-monitor her progress. [↑](#footnote-ref-3)
4. Directly provide the expectant woman encouragement, emotional support or practical help to perform the behavior. [↑](#footnote-ref-4)
5. Suggest or assist the expectant woman to seek encouragement, emotional support or practical help to perform the behavior from a friend, relative, colleague, or group. [↑](#footnote-ref-5)
6. Provide written, verbal or visual information about the behavior’s health, emotional, social or environmental consequences; encourage her to assess her feelings after attempts to perform the behavior; raise her awareness of future regret about performing the unwanted behavior. [↑](#footnote-ref-6)
7. Provide information or instruction to shape the expectant woman’s knowledge of *how to perform the behavior*. This includes identification of behavioral ‘triggers’ and their perceived causes. ‘Triggers’ are thoughts or situations that lead to performance of the unwanted behavior. [↑](#footnote-ref-7)
8. Change or support change of the expectant woman’s physical or social surroundings to facilitate performing the behavior, create barriers to an unwanted behavior, or avoid cues to an unwanted behavior. [↑](#footnote-ref-8)
9. Demonstrate the behavior; provide an observable example of the behavior; draw attention to others’ performance of the behavior as a model. [↑](#footnote-ref-9)
10. Identify, introduce, or alter social or environmental prompts or cues to promote the wanted behavior. [↑](#footnote-ref-10)
11. Identify, alter, or remove social or environmental prompts or cues to deter the unwanted behavior. [↑](#footnote-ref-11)
12. Encourage the expectant woman to practice performing the behavior or substitute it for an unwanted behavior. [↑](#footnote-ref-12)
13. Encourage the expectant woman to compare the pros and cons of changing the behavior, or to compare the outcomes of changing versus not changing the behavior. Includes encouraging the expectant woman’s imagination or observation of either the consequences of the unwanted behavior or rewards for the wanted behavior [↑](#footnote-ref-13)
14. Present verbal or visual communication from a credible source in favor of or against the behavior. [↑](#footnote-ref-14)
15. Provide or arrange for the expectant woman to receive a *material incentive or reward* (something of value) or a *social incentive or reward* (words of congratulation), or removal of an unpleasant consequence for making progress in performing the behavior. Includes encouraging the expectant woman to use self-incentives or self-rewards. [↑](#footnote-ref-15)
16. Use a threat of future punishment or removal of a reward as a consequence of performance of an unwanted behavior; arrange for a negative consequence or punishment following performance of an unwanted behavior. [↑](#footnote-ref-16)
17. Suggest strategies to minimize demands on the expectant woman’s mental resources to make it easier for her to perform the behavior. [↑](#footnote-ref-17)
18. Suggest to the expectant woman that performing the behavior might serve as an example to others. [↑](#footnote-ref-18)
19. Assist the expectant woman to identify discrepancies between her behavior and her values or self-image; encourage her to self-identify as someone who *used to* perform the unwanted behavior; suggest her adopting a new perspective to change thoughts or emotions about the behavior. [↑](#footnote-ref-19)
20. Promote the expectant woman’s self-belief that she can successfully perform the behavior, for example by persuading her about her capabilities and encouraging her to mentally rehearse success, focus on past success or use positive self-talk. [↑](#footnote-ref-20)
21. Provide referral or information to link the expectant woman to a community resource to assist in performing the behavior; review progress in completing the referral; support connections in completing the referral or perform an interagency case review. [↑](#footnote-ref-21)
22. Review the expectant woman’s experience accessing community resources to help her perform the behavior; assist in overcoming barriers to completing a referral. [↑](#footnote-ref-22)
23. Ask about and act on the expectant woman’s ideas on how to assist her in adhering to guidance from other providers regarding performing the behavior. [↑](#footnote-ref-23)
24. Gather information about the expectant woman’s readiness to change the behavior. [↑](#footnote-ref-24)
25. Assist the expectant woman to: set a behavior change goal; develop a plan to meet the goal using strategies to overcome barriers and increase facilitators; review her progress toward the goal; modify the goal or plan as needed. [↑](#footnote-ref-25)
26. Monitor the expectant woman’s progress in changing the behavior; give feedback on that progress; establish ways for the expectant woman to self-monitor her progress. [↑](#footnote-ref-26)
27. Directly provide the expectant woman encouragement, emotional support or practical help to perform the behavior. [↑](#footnote-ref-27)
28. Suggest or assist the expectant woman to seek encouragement, emotional support or practical help to perform the behavior from a friend, relative, colleague, or group. [↑](#footnote-ref-28)
29. Provide written, verbal or visual information about the behavior’s health, emotional, social or environmental consequences; encourage her to assess her feelings after attempts to perform the behavior; raise her awareness of future regret about performing the unwanted behavior. [↑](#footnote-ref-29)
30. Provide information or instruction to shape the expectant woman’s knowledge of *how to perform the behavior*. This includes identification of behavioral ‘triggers’ and their perceived causes. ‘Triggers’ are thoughts or situations that lead to performance of the unwanted behavior. [↑](#footnote-ref-30)
31. Change or support change of the expectant woman’s physical or social surroundings to facilitate performing the behavior, create barriers to an unwanted behavior, or avoid cues to an unwanted behavior. [↑](#footnote-ref-31)
32. Demonstrate the behavior; provide an observable example of the behavior; draw attention to others’ performance of the behavior as a model. [↑](#footnote-ref-32)
33. Identify, introduce, or alter social or environmental prompts or cues to promote the wanted behavior. [↑](#footnote-ref-33)
34. Identify, alter, or remove social or environmental prompts or cues to deter the unwanted behavior. [↑](#footnote-ref-34)
35. Encourage the expectant woman to practice performing the behavior or substitute it for an unwanted behavior. [↑](#footnote-ref-35)
36. Encourage the expectant woman to compare the pros and cons of changing the behavior, or to compare the outcomes of changing versus not changing the behavior. Includes encouraging the expectant woman’s imagination or observation of either the consequences of the unwanted behavior or rewards for the wanted behavior [↑](#footnote-ref-36)
37. Present verbal or visual communication from a credible source in favor of or against the behavior. [↑](#footnote-ref-37)
38. Provide or arrange for the expectant woman to receive a *material incentive or reward* (something of value) or a *social incentive or reward* (words of congratulation), or removal of an unpleasant consequence for making progress in performing the behavior. Includes encouraging the expectant woman to use self-incentives or self-rewards. [↑](#footnote-ref-38)
39. Use a threat of future punishment or removal of a reward as a consequence of performance of an unwanted behavior; arrange for a negative consequence or punishment following performance of an unwanted behavior. [↑](#footnote-ref-39)
40. Suggest strategies to minimize demands on the expectant woman’s mental resources to make it easier for her to perform the behavior. [↑](#footnote-ref-40)
41. Suggest to the expectant woman that performing the behavior might serve as an example to others. [↑](#footnote-ref-41)
42. Assist the expectant woman to identify discrepancies between her behavior and her values or self-image; encourage her to self-identify as someone who *used to* perform the unwanted behavior; suggest her adopting a new perspective to change thoughts or emotions about the behavior. [↑](#footnote-ref-42)
43. Promote the expectant woman’s self-belief that she can successfully perform the behavior, for example by persuading her about her capabilities and encouraging her to mentally rehearse success, focus on past success or use positive self-talk. [↑](#footnote-ref-43)
44. Provide referral or information to link the expectant woman to a community resource to assist in performing the behavior; review progress in completing the referral; support connections in completing the referral or perform an interagency case review. [↑](#footnote-ref-44)
45. Review the expectant woman’s experience accessing community resources to help her perform the behavior; assist in overcoming barriers to completing a referral. [↑](#footnote-ref-45)
46. Ask about and act on the expectant woman’s ideas on how to assist her in adhering to guidance from other providers regarding performing the behavior. [↑](#footnote-ref-46)
47. Gather information about the expectant woman’s readiness to change the behavior. [↑](#footnote-ref-47)
48. Assist the expectant woman to: set a behavior change goal; develop a plan to meet the goal using strategies to overcome barriers and increase facilitators; review her progress toward the goal; modify the goal or plan as needed. [↑](#footnote-ref-48)
49. Monitor the expectant woman’s progress in changing the behavior; give feedback on that progress; establish ways for the expectant woman to self-monitor her progress. [↑](#footnote-ref-49)
50. Directly provide the expectant woman encouragement, emotional support or practical help to perform the behavior. [↑](#footnote-ref-50)
51. Suggest or assist the expectant woman to seek encouragement, emotional support or practical help to perform the behavior from a friend, relative, colleague, or group. [↑](#footnote-ref-51)
52. Provide written, verbal or visual information about the behavior’s health, emotional, social or environmental consequences; encourage her to assess her feelings after attempts to perform the behavior; raise her awareness of future regret about performing the unwanted behavior. [↑](#footnote-ref-52)
53. Provide information or instruction to shape the expectant woman’s knowledge of *how to perform the behavior*. This includes identification of behavioral ‘triggers’ and their perceived causes. ‘Triggers’ are thoughts or situations that lead to performance of the unwanted behavior. [↑](#footnote-ref-53)
54. Change or support change of the expectant woman’s physical or social surroundings to facilitate performing the behavior, create barriers to an unwanted behavior, or avoid cues to an unwanted behavior. [↑](#footnote-ref-54)
55. Demonstrate the behavior; provide an observable example of the behavior; draw attention to others’ performance of the behavior as a model. [↑](#footnote-ref-55)
56. Identify, introduce, or alter social or environmental prompts or cues to promote the wanted behavior. [↑](#footnote-ref-56)
57. Identify, alter, or remove social or environmental prompts or cues to deter the unwanted behavior. [↑](#footnote-ref-57)
58. Encourage the expectant woman to practice performing the behavior or substitute it for an unwanted behavior. [↑](#footnote-ref-58)
59. Encourage the expectant woman to compare the pros and cons of changing the behavior, or to compare the outcomes of changing versus not changing the behavior. Includes encouraging the expectant woman’s imagination or observation of either the consequences of the unwanted behavior or rewards for the wanted behavior [↑](#footnote-ref-59)
60. Present verbal or visual communication from a credible source in favor of or against the behavior. [↑](#footnote-ref-60)
61. Provide or arrange for the expectant woman to receive a *material incentive or reward* (something of value) or a *social incentive or reward* (words of congratulation), or removal of an unpleasant consequence for making progress in performing the behavior. Includes encouraging the expectant woman to use self-incentives or self-rewards. [↑](#footnote-ref-61)
62. Use a threat of future punishment or removal of a reward as a consequence of performance of an unwanted behavior; arrange for a negative consequence or punishment following performance of an unwanted behavior. [↑](#footnote-ref-62)
63. Suggest strategies to minimize demands on the expectant woman’s mental resources to make it easier for her to perform the behavior. [↑](#footnote-ref-63)
64. Suggest to the expectant woman that performing the behavior might serve as an example to others. [↑](#footnote-ref-64)
65. Assist the expectant woman to identify discrepancies between her behavior and her values or self-image; encourage her to self-identify as someone who *used to* perform the unwanted behavior; suggest her adopting a new perspective to change thoughts or emotions about the behavior. [↑](#footnote-ref-65)
66. Promote the expectant woman’s self-belief that she can successfully perform the behavior, for example by persuading her about her capabilities and encouraging her to mentally rehearse success, focus on past success or use positive self-talk. [↑](#footnote-ref-66)
67. Provide referral or information to link the expectant woman to a community resource to assist in performing the behavior; review progress in completing the referral; support connections in completing the referral or perform an interagency case review. [↑](#footnote-ref-67)
68. Review the expectant woman’s experience accessing community resources to help her perform the behavior; assist in overcoming barriers to completing a referral. [↑](#footnote-ref-68)
69. Ask about and act on the expectant woman’s ideas on how to assist her in adhering to guidance from other providers regarding performing the behavior. [↑](#footnote-ref-69)
70. Gather information about the expectant woman’s readiness to change the behavior. [↑](#footnote-ref-70)
71. Assist the expectant woman to: set a behavior change goal; develop a plan to meet the goal using strategies to overcome barriers and increase facilitators; review her progress toward the goal; modify the goal or plan as needed. [↑](#footnote-ref-71)
72. Monitor the expectant woman’s progress in changing the behavior; give feedback on that progress; establish ways for the expectant woman to self-monitor her progress. [↑](#footnote-ref-72)
73. Directly provide the expectant woman encouragement, emotional support or practical help to perform the behavior. [↑](#footnote-ref-73)
74. Suggest or assist the expectant woman to seek encouragement, emotional support or practical help to perform the behavior from a friend, relative, colleague, or group. [↑](#footnote-ref-74)
75. Provide written, verbal or visual information about the behavior’s health, emotional, social or environmental consequences; encourage her to assess her feelings after attempts to perform the behavior; raise her awareness of future regret about performing the unwanted behavior. [↑](#footnote-ref-75)
76. Provide information or instruction to shape the expectant woman’s knowledge of *how to perform the behavior*. This includes identification of behavioral ‘triggers’ and their perceived causes. ‘Triggers’ are thoughts or situations that lead to performance of the unwanted behavior. [↑](#footnote-ref-76)
77. Change or support change of the expectant woman’s physical or social surroundings to facilitate performing the behavior, create barriers to an unwanted behavior, or avoid cues to an unwanted behavior. [↑](#footnote-ref-77)
78. Demonstrate the behavior; provide an observable example of the behavior; draw attention to others’ performance of the behavior as a model. [↑](#footnote-ref-78)
79. Identify, introduce, or alter social or environmental prompts or cues to promote the wanted behavior. [↑](#footnote-ref-79)
80. Identify, alter, or remove social or environmental prompts or cues to deter the unwanted behavior. [↑](#footnote-ref-80)
81. Encourage the expectant woman to practice performing the behavior or substitute it for an unwanted behavior. [↑](#footnote-ref-81)
82. Encourage the expectant woman to compare the pros and cons of changing the behavior, or to compare the outcomes of changing versus not changing the behavior. Includes encouraging the expectant woman’s imagination or observation of either the consequences of the unwanted behavior or rewards for the wanted behavior [↑](#footnote-ref-82)
83. Present verbal or visual communication from a credible source in favor of or against the behavior. [↑](#footnote-ref-83)
84. Provide or arrange for the expectant woman to receive a *material incentive or reward* (something of value) or a *social incentive or reward* (words of congratulation), or removal of an unpleasant consequence for making progress in performing the behavior. Includes encouraging the expectant woman to use self-incentives or self-rewards. [↑](#footnote-ref-84)
85. Use a threat of future punishment or removal of a reward as a consequence of performance of an unwanted behavior; arrange for a negative consequence or punishment following performance of an unwanted behavior. [↑](#footnote-ref-85)
86. Suggest strategies to minimize demands on the expectant woman’s mental resources to make it easier for her to perform the behavior. [↑](#footnote-ref-86)
87. Suggest to the expectant woman that performing the behavior might serve as an example to others. [↑](#footnote-ref-87)
88. Assist the expectant woman to identify discrepancies between her behavior and her values or self-image; encourage her to self-identify as someone who *used to* perform the unwanted behavior; suggest her adopting a new perspective to change thoughts or emotions about the behavior. [↑](#footnote-ref-88)
89. Promote the expectant woman’s self-belief that she can successfully perform the behavior, for example by persuading her about her capabilities and encouraging her to mentally rehearse success, focus on past success or use positive self-talk. [↑](#footnote-ref-89)
90. Provide referral or information to link the expectant woman to a community resource to assist in performing the behavior; review progress in completing the referral; support connections in completing the referral or perform an interagency case review. [↑](#footnote-ref-90)
91. Review the expectant woman’s experience accessing community resources to help her perform the behavior; assist in overcoming barriers to completing a referral. [↑](#footnote-ref-91)
92. Ask about and act on the expectant woman’s ideas on how to assist her in adhering to guidance from other providers regarding performing the behavior. [↑](#footnote-ref-92)
